# Six-Minute Activity-95^th^ Centile, a Novel Wearable-Derived Clinical Outcome Assessment for Duchenne Muscular Dystrophy

**DOI:** 10.1016/j.pediatrneurol.2025.11.017

**Published:** 2025-11-29

**Authors:** Nicholas Joy, Jonathan Soslow, W. Bryan Burnette, Andrew Liu, Christine C. Guo, Rakesh Pilkar, James C. Slaughter, Meng Xu, Kimberly Crum, Karry Su, Christopher Spurney, Nazia Husain, Katheryn Gambetta, Brian D. Soriano, Frank J. Raucci, Kan Hor, Larry W. Markham, Jaclyn Tamaroff

**Affiliations:** a Division of Pediatric Cardiology, Department of Pediatrics, Vanderbilt University Medical Center, Nashville, Tennessee; b Division of Pediatric Neurology, Department of Pediatrics, Vanderbilt University Medical Center, Nashville, Tennessee; c Ametris, L.L.C., Pensacola, Florida; d Department of Biostatistics, Vanderbilt University Medical Center, Nashville, Tennessee; e Children’s National Heart Institute, Children’s National Hospital, Washington, District of Columbia; f Division of Cardiology, Department of Pediatrics, Ann & Robert H. Lurie Children’s Hospital of Chicago, Northwestern University Feinberg School of Medicine, Chicago, Illinois; g Division of Cardiology, Department of Pediatrics, Seattle Children’s Hospital, University of Washington School of Medicine, Seattle, Washington; h Division of Cardiology, Department of Pediatrics, Children’s Hospital of Richmond at VCU, Richmond, Virginia; i Division of Pediatric Cardiology, Department of Pediatrics, The Ohio State University College of Medicine, Columbus, Ohio; j Division of Pediatric Cardiology, Department of Pediatrics, Indiana University School of Medicine, Indianapolis, Indiana; k Division of Pediatric Endocrinology and Diabetes, Department of Pediatrics, Vanderbilt University Medical Center, Nashville, Tennessee

**Keywords:** Duchenne muscular dystrophy, Ambulation, Clinical outcome assessment, Accelerometry

## Abstract

**Background:**

The six-minute walk test and quantitative muscle testing (QMT) are commonly used skeletal muscle assessments in Duchenne muscular dystrophy; however, they present challenges in nonambulatory patients. Our objective was to evaluate whether 6-min activity-95^th^ centile, a novel accelerometry metric capturing a participant’s greatest amount of movement in six consecutive minutes, distinguished between ambulatory and nonambulatory individuals and correlated with QMT data.

**Methods:**

Participants (N = 139) in an observational, longitudinal natural history study with median age of 12.0 years [interquartile range 10.0, 15.0] completed muscle testing and were instructed to wear an accelerometer on dominant wrist for 7 days and nights (a “wear”) at each of three annual study visits. One hundred two male participants were analyzed with a total of 184 valid wear periods.

**Results:**

Six-minute activity centiles declined over 2 years (n = 28, *P* < 0.001). No significant declines in centiles were detected immediately following loss of ambulation (n = 11). Significant correlations were observed between 6-min activity centiles and indexed QMT, with strongest at 95^th^ centile (r_s_ = 0.647, *P* < 0.001). There was a relationship between 6-min centiles and time since loss of ambulation.

**Conclusions:**

Ambulatory and nonambulatory patients with Duchenne muscular dystrophy were differentiated by 6-min activity and declined over time, modeling progression of skeletal myopathy. Six-minute activity-95^th^ centile has potential for future use as an effort-independent outcome of skeletal muscle progression and functional decline for both ambulatory and nonambulatory individuals.

## Introduction

Duchenne muscular dystrophy (DMD) is an X-linked recessive disorder due to a mutation in *DMD*, the gene encoding the protein dystrophin.^1^ Primarily affecting males, DMD has an estimated global incidence of one in every 3500–5000 male births and is characterized by its progressive nature.^2^ DMD leads to skeletal and cardiac muscle wasting and eventual cardiopulmonary failure and premature death with a median age of survival around 27 years (although the range varies from the teens to 40s).^3–6^ While there is no cure for DMD, improvements in cardiopulmonary support have increased the expected lifespan. In 2023, the first gene therapy for DMD received approval from the Food and Drug Administration, with expanded approval granted in 2024 to patients over 4 years of age, excluding those with exon 8 or 9 mutations, including nonambulatory patients. Due to this changing landscape, there is an emphasized need for reliable clinical outcomes assessment to measure and monitor skeletal muscle progression across the spectrum of disease.^7–10^ The North Star Ambulatory Assessment (NSAA) and six-minute walk test (6MWT) have previously shown significant correlations with accelerometry in individuals with DMD; however, use of both assessments is limited to ambulatory patients.^5,11^

Quantitative muscle testing (QMT) has been shown to provide an objective clinical measure of muscle strength and force and is suitable for use in nonambulatory patients.^11,12^ The test consists of force measurements in flexion and extension of both the upper and lower limbs. QMT has been shown to correlate with physical measures in patients with DMD including the NSAA and 6MWT.^11^ However, QMT is dependent on the effort, motivation, and state of the patient at the point of their testing. Corticosteroids have been utilized to preserve muscle strength and slow progression, which could have a significant effect on strength-dependent measures, such as QMT.^13^ In addition, as fat infiltration of skeletal muscle tissue contributes to declines in muscle strength, there may be a significant association between individual QMT score and user body mass index (BMI). Previous studies have investigated the relationship between QMT indexed to age (indexed QMT) and activity counts per minute utilizing accelerometry, particularly between indexed total arm QMT and wrist activity counts.^13–15^ While activity counts per minute can better capture skeletal muscle activity in a free-living manner, there are potential drawbacks to using this metric as it can be subject to overestimation in average count value. It would be advantageous to have a reliably correlated effort-independent measure in free-living of skeletal muscle disease progression for both ambulatory and nonambulatory patients.

Six-minute activity-95^th^ centile (6M95c) is a novel accelerometry-derived measure of peak physical activity to mirror the concept of interest assessed by the 6MWT but in a free-living environment. To derive this metric, we first calculated the sum of activity counts, measured as vector magnitudes (VMs), of each consecutive 6-min epochs when patients wear the devices during the day and then derived the 95^th^ centile of these VM sums of all 6-min epochs. The 95^th^ centile was preferentially selected as the measure of peak activity over centiles such as the 90^th^ or 99^th^ inline with the validated measure stride velocity 95^th^ centile, an ankle accelerometer-derived endpoint validated in populations with DMD.^16^ The strength of 6M95c is that it measures peak activity with respect to the individual wearing the accelerometer during free-living passively. Therefore, 6M95c is not effort-dependent and may better represent a participant’s disease progression when compared to strength assessment measured at a single point in time. The aim of this pilot study was to assess the correlation between indexed QMT score and 6M95c in individuals with DMD. A secondary objective was to utilize multiple 6-min activity centiles to model a longitudinal pattern in muscular disease progression and loss of ambulation.

## Materials and Methods

### Participants

The data used in this analysis come from a larger ongoing longitudinal natural history study of individuals with DMD. A cohort of 139 individuals with DMD of at least 8 years of age was enrolled. Participants were enrolled from Vanderbilt University Medical Center (N = 39), Nationwide Children’s Hospital (N = 43), Riley Children’s Hospital (N = 16), Children’s National Medical Center (N = 17), Lurie Children’s Hospital (N = 10), Seattle Children’s Hospital (N = 1), and Children’s Hospital of Richmond at VCU (N = 13). Participant-reported demographics were obtained, and demographics of those included for analysis were reported (Table 1). Medications, including use of glucocorticoids, and medical history, including ambulatory status and mutation of *DMD* gene, were documented (Table 1).^17^ Participant ambulatory status was captured as part of skeletal muscle assessment. Height and weight were measured on the date of the annual study visit. BMI z-scores were calculated using CDC 2000 growth charts.^18^ This study was approved by the Vanderbilt Institutional Review Board (IRB 181942). All procedures were performed in compliance with relevant laws and institutional guidelines. Participants over the age of 18 years provided written informed consent, while participants under the age of 18 years provided assent and parent or guardian informed consent. Privacy rights of human subjects were observed.

### Quantitative muscle testing

QMT was performed during each study visit using a handheld myometer as previously described.^5^ Total arm scores were the sum of elbow flexion and extension (pounds) scores for both arms. Total leg scores were the sum of knee flexion and extension scores from both knees. Total QMT was the sum of total arm and total leg. Scores were indexed according to age to account for progressive loss in skeletal muscle strength associated with DMD, as previously described.^11,13^ Healthy male children show demonstrate linear increase in QMT up until 20 years of age, where strength is generally maintained. In males with DMD, strength instead declines yearly, so indexing QMT scores aims to better reflect the disease’s severity.

### Six minute activity centiles

Triaxial accelerometers (GT9X, Ametris, Pensacola, FL) were utilized to measure 6 minute activity. Participants were instructed to wear an accelerometer on the wrist of their dominant hand for 7 days, 24 hours a day, (quantified as a “wear”) only removing to bathe or swim (Supplementary Figs 1 and 2). Accelerometry has been shown to be consistent between upper and lower limb, indicating the possible need of just one accelerometer for measurements, rather than requiring both wrist and ankle accelerometry data.^13^ Accelerometry data collection began immediately after study visit. Data were collected at a frequency of 30 Hz (30 data points per second per axis) and were integrated into 15 second epochs.

**FIGURE 1.**
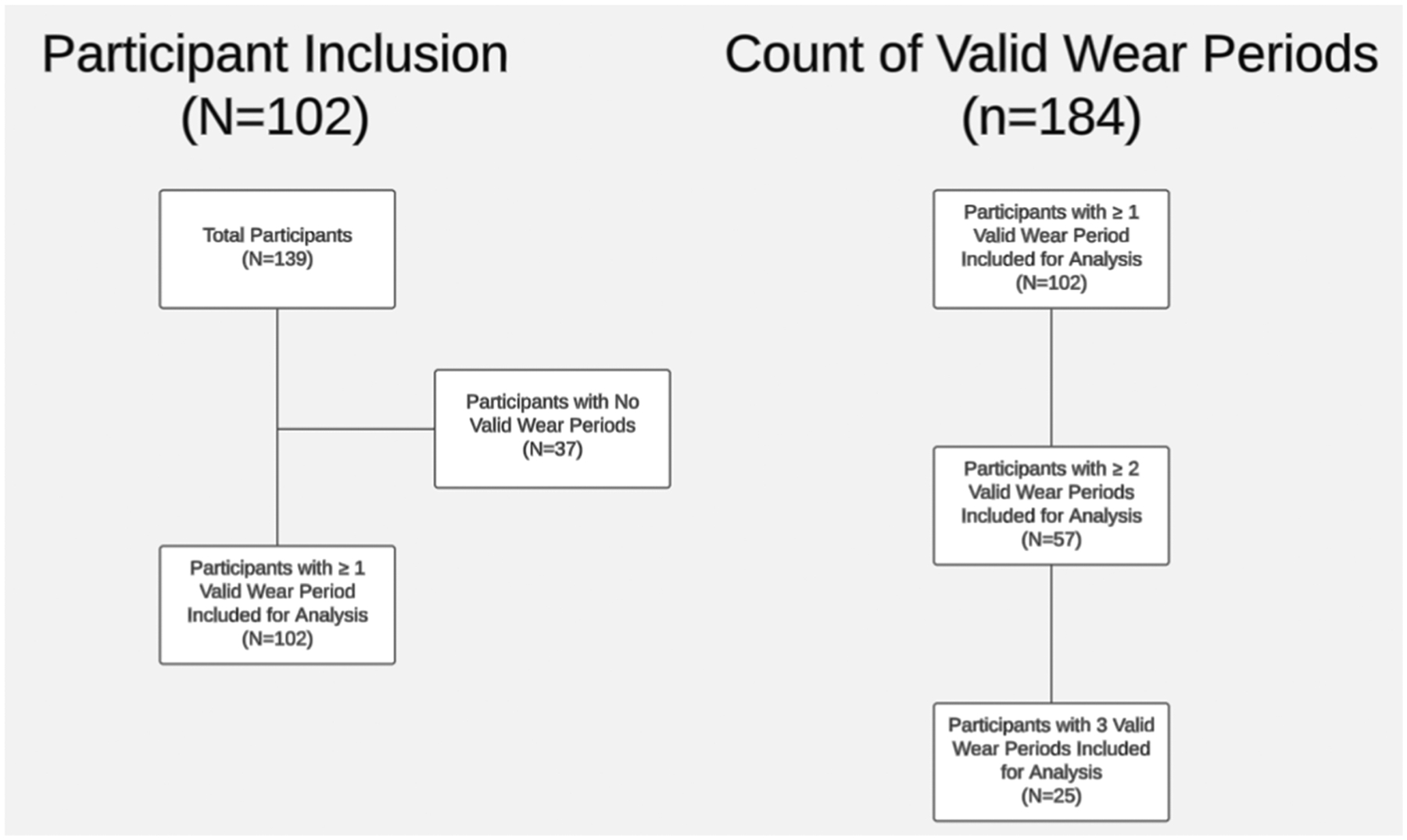
Determination of valid wear data. N = 37 participants were excluded from analysis due to having no valid accelerometry wear data available. From the N = 102 participants with valid wear periods, a total of 184 valid wear periods were analyzed.

The raw data were converted to VM counts using ActiLife software (Ametris, Pensacola, FL) by taking the square root of the sum of the three axial measures squared. Participants with a wear period of less than 3 days of valid wear time were excluded from analysis (Fig 1).^19,20^ For purposes of analysis, a valid day consisted of at least 600 minutes of wear between 7am and 10pm (“daytime”) and at least 300 minutes of wear between 10pm and 7am (“overnight”).

**FIGURE 2.**
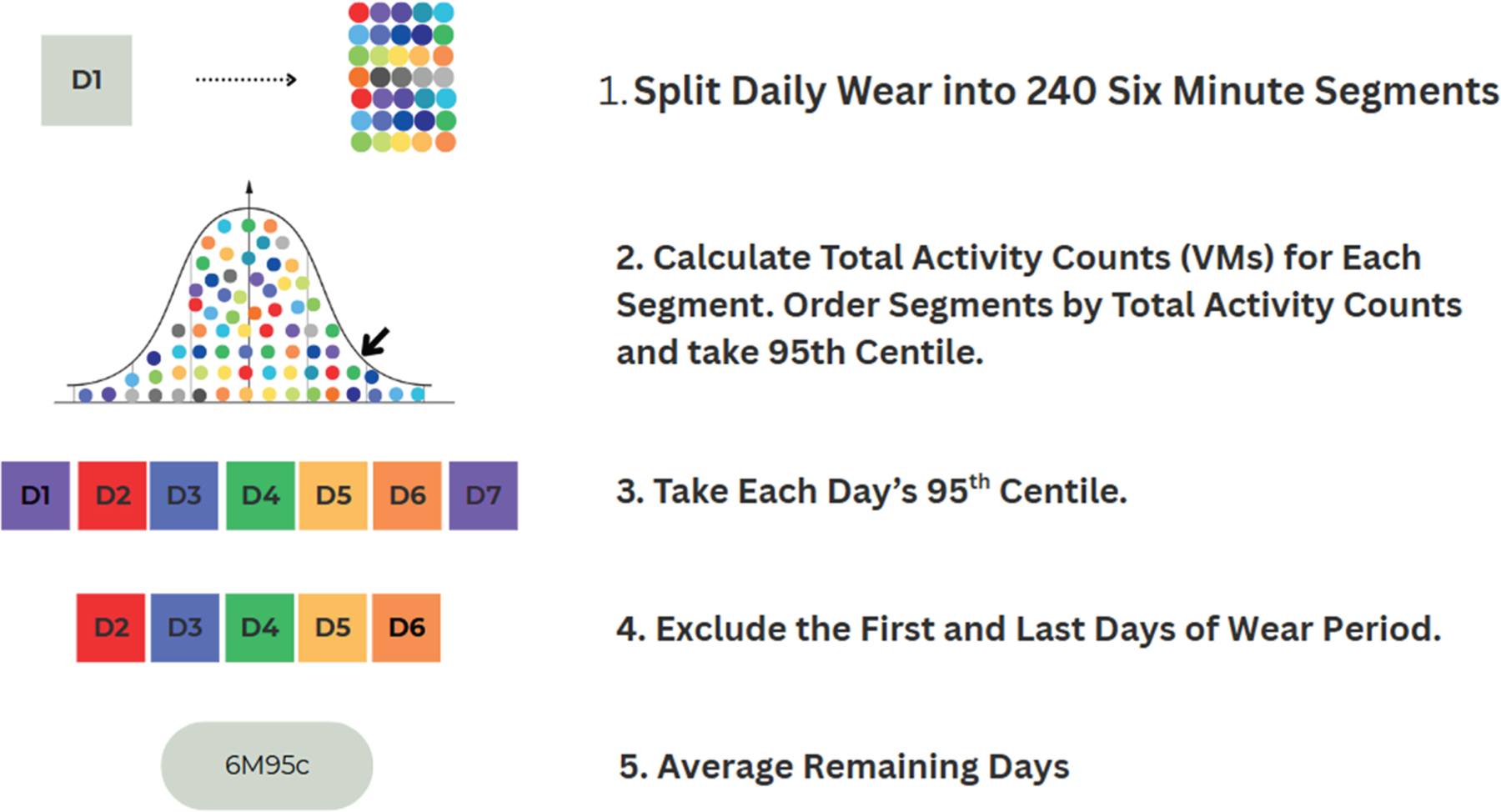
Visual representation of computing 6M95c. 6M95c = six-minute activity-95^th^ centile. The color version of this figure is available in the online edition.

Six-minute activity was derived from activity counts measured as VMs by dividing total daily-midnight to midnight-wear into 6-min segments and taking the summed VMs of each segment. Daily 25^th^, 75^th^, 50^th^, and 95^th^ centiles of 6 minute activity were then averaged for each subject-excluding their first and last days of wear-to compute overall 25^th^, 50^th^, 75^th^, and 95^th^ centiles (Fig 2). The daily average 95^th^ centile (6M95c) was chosen as a measure of an individual’s peak activity.

### Cardiac magnetic resonance imaging

Cardiac magnetic resonance imaging (CMR) was carried out using a 1.5 Tesla scanner as previously described.^21^ Intravenous gadolinium contrast (gadopentate dimeglumine, Magnevist, Bayer Healthcare Pharmaceuticals, Wayne, NJ, at a dose of 0.2 mmol/kg or gadobutrol, Gadavist, Bayer Healthcare Pharmaceuticals, Wayne, NJ, at a dose of 0.15–0.2 mmol/kg) was administered through a peripheral intravenous line. CMR measures of interest collected included left ventricular ejection fraction (LVEF), global percentage late gadolinium enhancement (LGE), and global circumferential strain (Ecc).

### Statistical analysis

**FIGURE 3.**
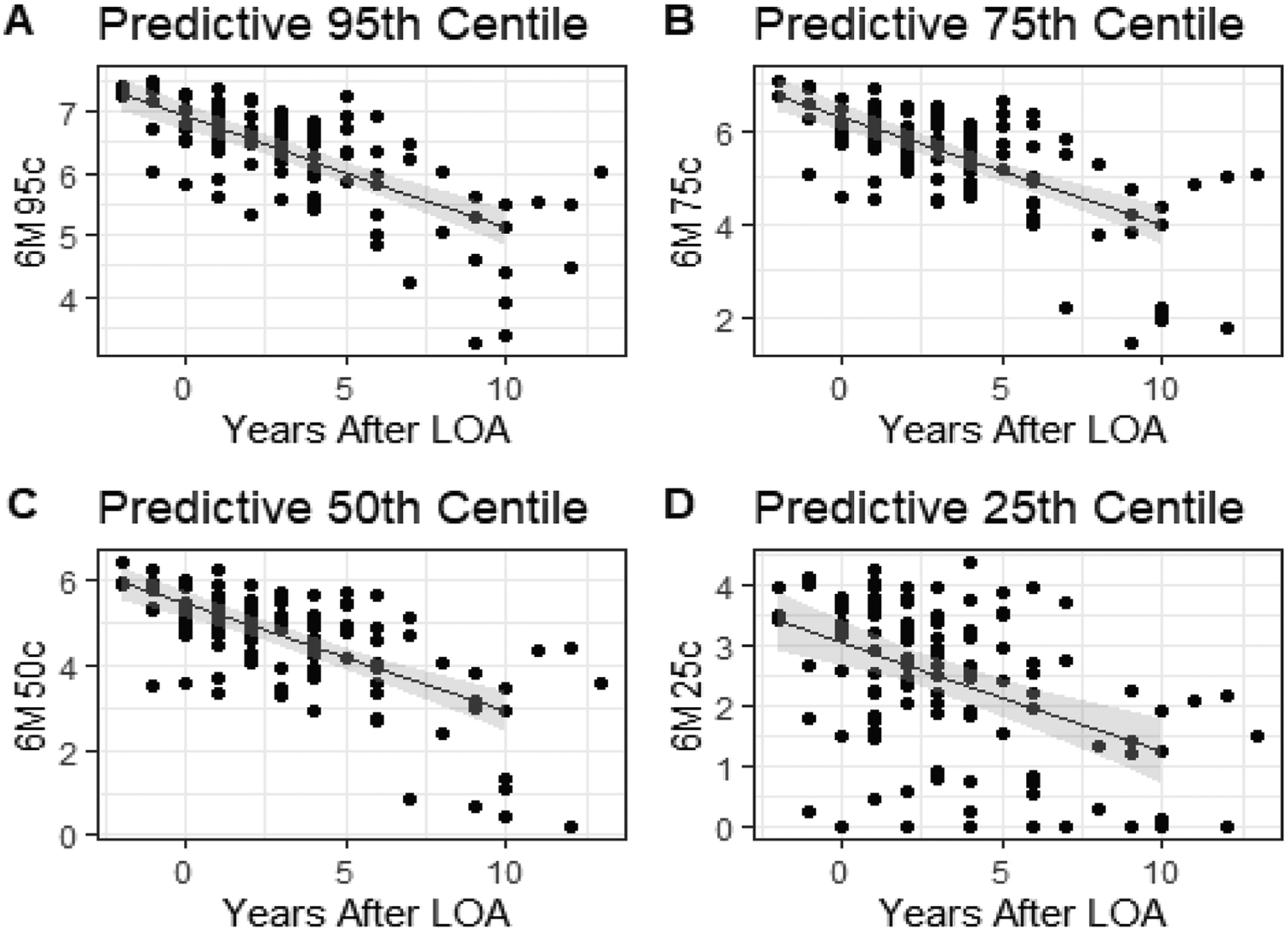
Regression of 6 minute activity centiles against time (years) since loss of ambulation (N = 63 participants, n = 116 wears). Best-fit regression line plotted against individual nonambulatory wear periods. (A) Regression of 6M95c against time since loss of ambulation. (B) Regression of 6M75c against time since loss of ambulation. (C) Regression of 6M50c against time since loss of ambulation. (D) Regression of 6M25c against time since loss of ambulation. 6M95c = six-minute activity-95^th^ centile. 6M75c = six-minute activity-75^th^ centile; 6M50c = six-minute activity-50^th^ centile; 6M25c = six-minute activity-25^th^ centile.

**FIGURE 4.**
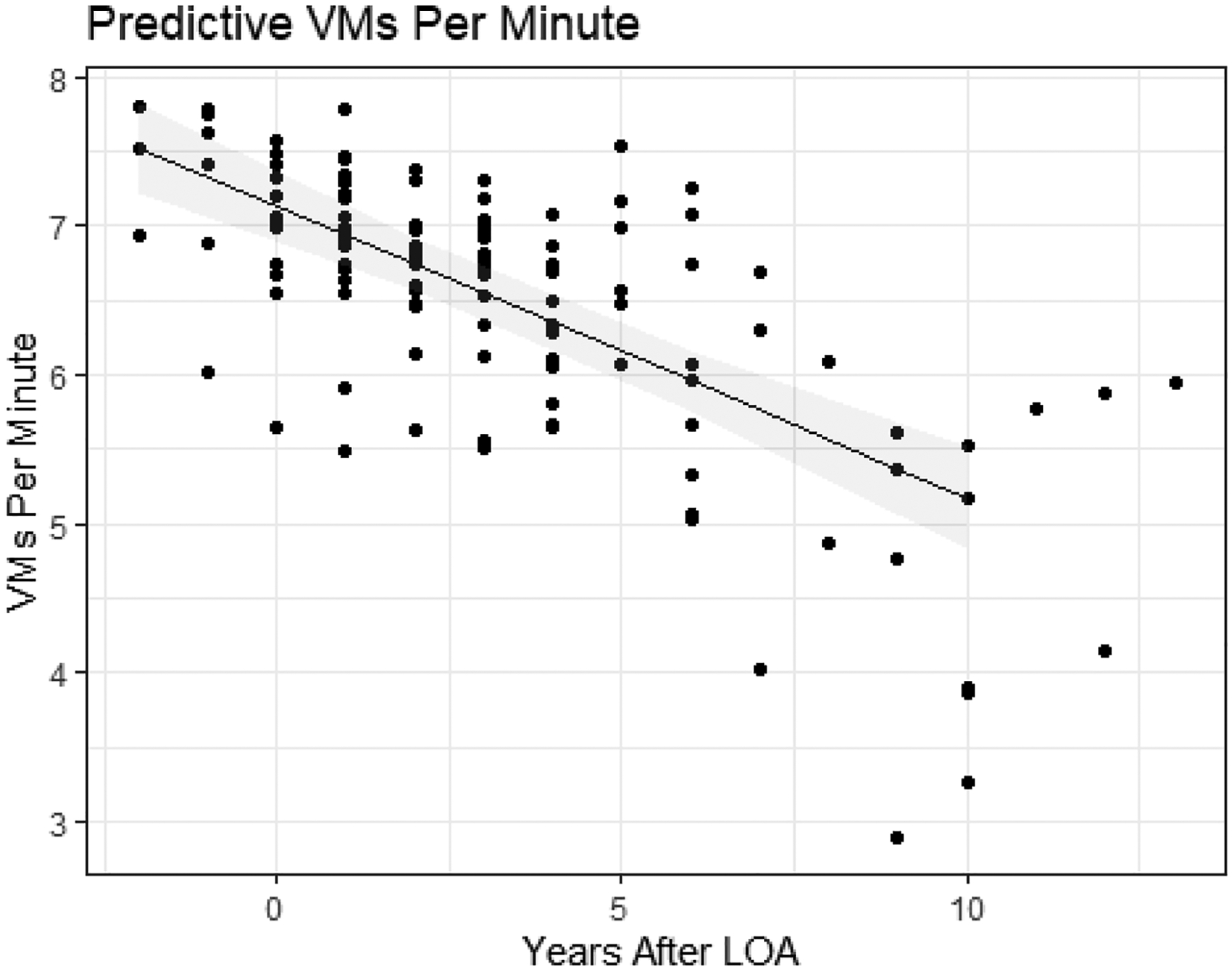
Regression of VMs per minute against time (years) since loss of ambulation (N = 63 participants, n = 116 wears). Best-fit regression line plotted against individual nonambulatory wear periods. VMs = vector magnitudes.

To assess whether 6 minute activity is able to detect a significant difference in activity between ambulatory and nonambulatory individuals, linear mixed effects models of the 25^th^, 50^th^, 75^th^, and 95^th^ centiles were formed controlling for multiple participant occurrences and assessing a fixed effect of ambulatory status (Table 2). To determine if there was a significant change in 6 minute activity over time and following loss of ambulation, Wilcoxon signed-rank tests of the 6 minute activity centiles were performed for visits at baseline versus 2-year follow-up, as well as immediately preceding and following loss of ambulation (Tables 3 and 4). When identifying the 6 minute activity centiles preceding and following an individual’s loss of ambulation, the accelerometry wears corresponding to the participant’s last visit assessed as ambulatory and first visit assessed to be nonambulatory were identified. In addition, 6 minute activity 25^th^, 50^th^, 75^th^, and 95^th^ centiles were regressed against the number of years preceding or following a participant’s loss of ambulation, and participant BMI z-score and duration of corticosteroid use were added to test any significant influence (Table 5, Figs 3 and 4). To evaluate the relationship between 6 minute activity and QMT, Spearman correlations were computed to assess associations of 6 minute activity centiles and indexed QMT metrics (Table 6). Duration of corticosteroid use (years) and participant BMI z-score were then added to this model to determine any significant effect. For participants with available CMR data at time of accelerometer placement, Spearman correlations were drawn between 6 minute activity centiles and measures of LVEF, LGE, and Ecc.

Statistical analyses were carried out using R (version 4.2.3; R Core Team, 2023), the *sasLM* (v0.10.2; Bae, 2024) and the *summarytools* (v1.0.1; Comtois, 2022) packages. The full reproducible code is available in the supplement (Supplementary Material 3). A level of *P* < 0.05 was established for significance, and correlation was described using thresholds of r < 0.4 for “weak”, 0.4< r < 0.7 for “moderate”, and r > 0.7 for “strong”.^22^

## Results

### Participant characteristics

Of the 139 participants, 102 males were included (Table 1). The median age of participants at baseline was 12.0 years old [interquartile range: 10.0, 15.0]. Ten participants were over the age of 18 years at baseline. About half (48%) of participants were ambulatory at baseline. There were 94 (92.2%) participants previously on or currently taking corticosteroids. Among participants with reported genetics (N = 88), a deletion mutation of the *DMD* gene was most common, with the most common deletion including exons 49–50. Thirty-seven participants had no valid wear periods and were excluded from the analysis (Fig 1). Of these excluded participants, 16 did not have any accelerometry data available due to either lost accelerometers or missed study visits, while 21 participants had low compliance in their wear period and failed to record the minimum 3 valid day threshold. We did not detect differences in demographic or anthropometric data between those included in the analysis and those without valid data.

### Analysis of 6-min activity

#### Ambulatory versus nonambulatory wears

A total of 184 valid wear periods were analyzed (Fig 1) from 102 individuals with 1–3 annual visits per person. Significant differences were noted in median 25^th^ (*P* < 0.001), 50^th^ (*P* < 0.001), 75^th^ (*P* < 0.001), and 95^th^ (*P* < 0.001) 6 minute activity centiles between all ambulatory and nonambulatory wears (Table 2).

#### Longitudinal decline in 6 minute activity

There were also significant differences in the median 50^th^ (*P* < 0.001), and 95^th^ (*P* < 0.001), 75^th^ (*P* < 0.001) centiles between baseline and 2 years follow-up for subjects with available 2-year follow-up wear data (N = 28) (Table 3).

#### Six minute activity in loss of ambulation

Eleven participants experienced loss of ambulation during the study. In this small sample, no significant differences were detected in median 25^th^, 50^th^, 75^th^, and 95^th^ centiles between the last ambulatory and first nonambulatory wear (Table 4).

#### Regression analyses

For individuals who were nonambulatory when wearing the accelerometer, 6 minute activity centiles and VMs per minute were regressed against the length of time (years) since loss of ambulation (Fig 2). The regressions were fitted to a log-linear mixed effects model, accounting for both the scale in magnitude of the activity centiles as well as repeated subject measures between study visits (Table 5). The linear model regressing 6M95c against time since loss of ambulation had the greatest proportion of variance explained by time since loss of ambulation (Table 5). Duration of corticosteroid use and BMI z-score had no significant effect on the relationship between 6M95c and time since loss of ambulation.

#### Correlations between 6 minute activity and QMT

For all wears with available QMT data (N = 97 participants, n = 168 wears), correlations were determined between 6 minute activity centiles and indexed total arm, leg, and overall QMT values (Table 6). Total and indexed QMT data is described in Table A.1. Correlations between VMs per minute and total arm, leg, and overall QMT values were also assessed (Table 6). The strongest correlations observed were between indexed total arm QMT and 6M95c (r_s_ = 0.65, *P* < 0.001), indexed total leg QMT and VMs per minute (r_s_ = 0.62, *P* < 0.001), and indexed overall QMT with 6M95c (r_s_ = 0.65, *P* < 0.001). These correlations were not significantly modified when adjusted for by participant BMI z-score and duration of corticosteroid use. Holm correction was done on *P* values, and all remained significant after correction (Table A.2).

#### Correlations between 6 minute activity and cardiac magnetic resonance imaging measures

For all wears with associated Cardiac magnetic resonance imaging data available (N = 81 participants, n = 127 wears), correlations were drawn between 6 minute activity centiles and LGE, LVEF, and Ecc. No significant correlations were found between 6 minute activity centiles and the measures (Table A.3).

## Discussion

In males with DMD, 6M95c, evaluated via accelerometry, was found to differentiate between ambulatory and nonambulatory individuals. In addition, 6M95c decreased significantly from baseline to 2-year follow-up. Moderately strong correlations were mapped between 6M95c and QMT measures.

No significant decline in 6M95c between the year immediately preceding and immediately following loss of ambulation was noted (Table 4). However, this may have been due to the relatively small number of participants who experienced loss of ambulation during the study (N = 11). Larger longitudinal studies of individuals with DMD encompassing the time of loss of ambulation are needed to assess the potential changes in 6M95c before and after loss of ambulation. In addition, the influences of participant BMI and corticosteroid use had no effect on the relationship between 6M95c and time since loss of ambulation.

The mixed effects model of 6M95c had both the highest proportion of variance explained by length of time since loss of ambulation, as well as the lowest explained by subject (Table 5). The strong fit of the regression model predicting 6M95c based on time since loss of ambulation supports its use as an outcomes measure of skeletal myopathy progression. High variance between subjects would imply the influence of the individual’s effort capacity in measuring their physical activity. With low variance between subjects in its model, 6M95c is less dependent on the effort of its wearer and able to assess the individual’s peak physical activity in free-living. Notably, each regression model includes a set of four data points which deflect above the predicted curve from years 10 through 13 after loss of ambulation (Fig 2). The data points come from multiple different participants and help to visualize small individual variations in the progression of skeletal myopathy for those with DMD. Further investigation of this model, including influence of participant genotype and variations in the type and location of mutation, is needed.

Indexed QMT is a commonly used metric to evaluate muscle strength in individuals with DMD and previous studies found a significant association between participant VMs per minute and indexed QMT.^13^ 6M95c has been found to have stronger correlations with indexed arm and total QMT compared to VMs per minute (Table 6) in this cohort. For 6M95c, a stronger predictive log-linear model for decline in peak skeletal muscle activity following loss of ambulation coupled with stronger QMT correlates and supports 6M95c as a new metric of physical accelerometry capable of measuring skeletal muscle progression in both ambulatory and nonambulatory patients with DMD.

For ambulatory patients with DMD, assessments including the NSAA and 6MWT remain standard clinical measures of skeletal muscle performance particularly useful in early stage monitoring of skeletal disease progression.^11^ In this pilot study, ambulatory participant 6MWT and NSAA data were not assessed. Future analysis of 6M95c′s correlation with standard clinical measures such as NSAA and 6MWT is needed to assess the accelerometry measure’s potential convergent validity as a measure of peak performance in ambulatory DMD populations. In addition, as 6M95c can be assessed in both ambulatory and nonambulatory DMD populations the measure has potential to complement ambulatory measures, like NSAA or 6MWT, and be able to bridge loss of ambulation and continue to provide a meaningful measure of skeletal muscle activity when nonambulatory.

In addition to indexed QMT, other novel upper limb skeletal muscle functional measures have been developed for nonambulatory patients with DMD which include performance of upper limb 2.0 (PUL 2.0 and the DMD Upper Limb [UL] patient-reported outcome measure, DMD UL PROM).^23,24^ While correlations between PUL 2.0 and DMD UL PROM have been drawn, there are no prior studies, to our knowledge, where either was shown to correlate with wrist accelerometry.^25^ One advantage to 6M95c is its objectivity in measuring an individual’s skeletal muscle performance through accelerometry, a measure which can be captured in free-living movements, rather than necessitating a clinical environment. This contrasts with the high subjectivity in measures PUL 2.0 and the DMD UL PROM from rater evaluation or patient self-report, respectively. One study found significant, nonlinear declines in PUL 2.0 total score over time, evidence of a nonlinear progression of skeletal myopathy with DMD which is also reflected in the log-adjusted declines in 6M95c for our cohort.^23^ Particularly as declines in upper limb activity have been shown to follow a proximal to distal progression, future studies analyzing the association between declines in PUL 2.0 or DMD UL PROM and declines in 6M95c are needed.^24,25^ Such studies could not only evidence the longitudinal decline in upper limb skeletal muscle function but demonstrate the effect of objectivity in measure of this function as well. In an additional analysis, correlations between 6M95c and cardiac magnetic resonance imaging metrics—including LVEF, LGE, and global circumferential strain—were nonsignificant (Table A.3). This finding is consistent with a previous study which found nonsignificant correlations between these same cardiac biomarkers and indexed limb QMT.^21^ As QMT is a validated clinical measure assessing an individual’s skeletal muscle performance, it makes sense that comparisons between limb QMT and measures of an individual’s cardiac function would not demonstrate a significant relationship. This is evidence for QMT’s discriminant validity as a skeletal muscle measure. Similarly, the significant correlations between 6M95c and indexed QMT support convergent validity of 6M95c with the clinical measure, while nonsignificant correlations with the cardiac function measures support discriminant validity of 6M95c from cardiac function. Taken together, these correlations are supportive evidence that 6M95c is a measure of skeletal muscle function, rather than cardiac.

### Limitations

The analyses of participants who completed year-two follow-up or experienced loss of ambulation during the study were limited by their small numbers of participants. The statistical significance was potentially weakened by these small sample sizes, limiting the ability to capture declines in skeletal muscle performance over the study’s duration as well as over loss of ambulation (Tables 3 and 4). Studies further evaluating 6M95c before and after loss of ambulation are needed. The gradual declines of 6M95c and other 6 minute activity centiles are apparent (Fig 2), deserving further investigation. This trend also reflects clinical presentation where the transition from ambulatory to nonambulatory status is gradual, not discrete.

Due to the requirement of at least three valid accelerometry wear days, 37 of the 139 participants were excluded from analysis. This is a large proportion of the cohort which could impact the validity of the results. However, statistical analysis found no significant differences in median baseline age, height, weight, or BMI between those included and those excluded. In addition, a chisquare contingency test found no significant association between the relative proportion of individuals who were ambulatory versus nonambulatory at baseline between the group of participants included in analysis and those excluded.

While demographic characteristics did not differ, better parameters are needed in future studies to ensure greater participant compliance in wear, a challenge faced by greater than half (56.8%) of the excluded 37 participants. A key advantage to accelerometer technology in patients with DMD is the ability to monitor an individual’s skeletal muscle performance remotely, however this advantage is limited by how compliant the individual is with wearing the device. Strategies to improve participant compliance with the accelerometer have been successfully incorporated in other studies. Reminder communication such as emails and scheduled text messages can aid participants in remembering to continue wearing the accelerometer device for the entire duration instructed and return the device once the wear period is complete. Incorporation of daily electronic surveys can be used to track time periods where the device was not worn, while serving as another reminder to wear the accelerometer. Lastly, for remote studies that include a financial incentive for its participants, waiting to extend the incentive until study materials have returned can facilitate smoother compliance with device delivery to and from the participant.

There are two limitations to note in the analysis of those who experienced loss of ambulation during the study. First, separation of the final ambulatory and first nonambulatory wear period was compared based on the year loss of ambulation was reported as more precise data for loss of ambulation is challenging to gather. Individual variations in study visit timing, therefore, are difficult to assess. Second, as the longitudinal models suggest (Fig 2), loss of ambulation is a gradual process for individuals meaning the timing of a significant decline in skeletal muscle performance may vary across participants, rather than consistently during the interval analyzed. Taken together, these limitations make comparisons across loss of ambulation less precise and could impact 6M95c′s ability to differentiate ambulatory and nonambulatory individuals in the immediate year both preceding and following loss of ambulation.

While the linear mixed-effect models can account for repeated measures of subjects’ accelerometer wear, the strength of these models are limited by the small number of wear periods available per subject. Most subjects contributed at most 2 to 3 valid wear periods to the regression models; this does limit the interpretability of between-subjects variance captured in the model. A future longitudinal study with many more wear periods available per user would be advantageous to further assess the relationship between 6M95c and time since loss of ambulation.

While the pilot study found that 6M95c differentiated across ambulatory status and showed longitudinal declines correlating with declines in upper limb skeletal muscle function, the findings are presently limited in the ability to validate 6M95c as a performance measure. Future study comparing 6M95c data from both ambulatory and nonambulatory participants with DMD with standardized 6M95c data from healthy controls is needed. In addition, the present 6M95c data is limited in the accelerometer’s ability to account for noise contributed by external sources of movement such as a powered wheelchair. This noise is certainly of interest as it could be a steady source of bias across both ambulatory and nonambulatory cohorts as the use of assistive devices in movement gradually increases before loss of ambulation. While this noise is typically not accounted for,^13,15,26–28^ further study is necessary to determine the extent of this external bias on 6M95c′s measure and create an adjustment factor for the noise.

## Conclusions

6M95c is a novel wearable-derived clinical outcome assessment that can measure skeletal muscle disease progression in individuals with DMD. Critically, it can be effectively evaluated in individuals who are both ambulatory and nonambulatory. 6M95c has significant, moderately strong associations with indexed QMT metrics, and declines with an increase in the number of years since loss of ambulation. While further studies in independent cohorts are needed, this pilot study utilizing 6M95c shows potential for the accelerometry-derived measure as a marker of skeletal muscle disease progression in individuals with DMD. In addition, this dataset has potential applications to serve as a baseline for comparison at endpoints of future clinical trials including gene therapy, where the free-living activity measure of 6M95c could be utilized as an effort-independent, at home performance outcome for therapy recipients.

## Supplementary Material

1

2

3

4

5

6

Supplementary data

Supplementary data related to this article can be found at https://doi.org/10.1016/j.pediatrneurol.2025.11.017.

## Figures and Tables

**TABLE 1.** Participant Characteristics at Baseline

Participants Characteristics (N = 102)	
	Median (IQR) or n (%)
Age (years)	12.0 (10.0, 15.0)
Height (cm)	142.2 (132.1, 154.0)
Weight (kg)	45.2 (33.7, 59.3)
BMI z-score	1.2 (−0.1, 1.8)
Taking/previously taken corticosteroids (%)	92.2%
Ambulatory (%)	48.0%

Race		Ethnicity		
	N (%)	Hispanic	Not Hispanic	Unknown/Did Not Provide
White	81 (79.4%)	5 (6.2%)	76 (93.8%)	0
African American	7 (6.9%)	0	7 (100%)	0
Asian	5 (4.9%)	0	5 (100%)	0
Multiracial	3 (2.9%)	0	3 (100%)	0
Unknown/did not provide	6 (5.9%)	3 (50.0%)	0	3 (50.0%)
Participant Genotypes (N = 88)				
Duplication mutation (%)				10.2%
Missense mutation (%)				6.8%
Nonsense mutation				9.1%
Deletion mutation (%)				73.9%

Abbreviations:

BMI = Body mass index

IQR = Interquartile range

**TABLE 2.** Six-Minute Activity Centiles for Ambulatory Versus Nonambulatory Individuals

Centile	Ambulatory 6 MA-VMs (n = 74)	IQR	Nonambulatory 6 MA-VMs (n = 110)	IQR	*P* Value for the Difference
6M25c	25.57	[17.83, 36.60]	14.12	[4.70, 31.56]	<0.001
6M50c	218.00	[171.10, 327.52]	134.85	[57.07, 204.55]	<0.001
6M75c	613.32	[490.02, 819.13]	324.88	[163.42, 457.82]	<0.001
6M95c	1404.61	[1160.22, 1609.70]	687.23	[405.19, 889.04]	<0.001

Abbreviations:

6M95c = Six-minute activity-95^th^ centile

6M75c = Six-minute activity-75^th^ centile

6M50c = Six-minute activity-50^th^ centile

6M25c = Six-minute activity-25^th^ centile

6MA = Six-minute activity

IQR = Interquartile range

VMs = Vector magnitudes

N = 102 Participants; n = 184 Wears.

Median differences in ambulatory and nonambulatory 6 MA (VMs/6 min segment) at the 25^th^, 50^th^,75^th^ and 95^th^ centiles.

**TABLE 3.** Six-Minute Activity Centiles at Baseline and Year Two Follow-Up (N = 28)

Centile	Baseline 6 MA-VMs (N = 28)	IQR	Year Two 6 MA-VMs (N = 28)	IQR	*P* Value for the Difference (Paired)
6M25c	25.95	[8.87, 36.97]	19.90	[6.07, 34.95]	0.368
6M50c	170.37	[107.04, 233.20]	149.00	[76.38, 212.05]	<0.001
6M75c	421.74	[265.88, 607.23]	357.41	[206.72, 461.10]	<0.001
6M95c	850.16	[591.26, 1296.01]	745.65	[389.10, 980.47]	<0.001

Abbreviations:

6M95c = Six-minute activity-95^th^ centile

6M75c = Six-minute activity-75^th^ centile

6M50c = Six-minute activity-50^th^ centile

6M25c = Six-minute activity-25^th^ centile

6MA = Six-minute activity

IQR = Interquartile range

VMs = Vector magnitudes

Median differences in baseline and 2-year follow-up 6 MA (VMs/6 min segment) at the 25^th^, 50^th^, 75^th^, and 95^th^ centiles.

**TABLE 4.** Six-Minute Activity Centiles Before and After Loss of Ambulation (N = 11)

Centile	Ambulatory 6 MA-VMs (N = 11)	IQR	Nonambulatory 6 MA-VMs (N = 11)	IQR	*P* Value for the Difference (Paired)
6M25c	22.23	[7.62, 54.07]	23.78	[3.94, 36.98]	0.148
6M50c	221.07	[107.26, 393.58]	195.86	[118.46, 238.56]	0.148
6M75c	553.42	[315.74, 882.31]	453.48	[296.54, 558.71]	0.067
6M95c	1306.66	[636.68, 1521.07]	898.75	[689.28, 1139.50]	0.083

Abbreviations:

6M95c = Six-minute activity-95^th^ centile

6M75c = Six-minute activity-75^th^ centile

6M50c = Six-minute activity-50^th^ centile

6M25c = Six-minute activity-25^th^ centile

6MA = Six-minute activity

IQR = Interquartile range

VMs = Vector magnitudes

Median differences pre-LOA and post-LOA 6 MA (VMs/6 min segment) at the 25^th^, 50^th,^ 75^th^, and 95^th^ centiles.

**TABLE 5.** Predictive Regression Models of Six-Minute Activity Centiles Based on Time (Years) Following Loss of Ambulation

Centile	Intercept [95% CI]	Slope [95% CI]	Subject Variance	Residual Variance	Marginal R^2^/Conditional R^2^
6M25c	3.04* [2.68, 3.40]	−0.18* [−0.26, - 0.10]	0.85	0.43	0.203/0.733
6M50c	5.43* [5.13, 5.73]	−0.25* [−0.31, - 0.19]	0.70	0.21	0.414/0.864
6M75c	6.29* [6.01, 6.57]	−0.23* [−0.28, - 0.18]	0.54	0.15	0.439/0.876
6M95c	6.91* [6.71, 7.11]	−0.18* [−0.22, - 0.14]	0.27	0.10	0.469/0.851
VMs per minute	7.13* [6.89, 7.37]	−0.20* [−0.24, - 0.16]	0.38	0.13	0.436/0.858

Abbreviations:

6M95c = Six-minute activity-95^th^ centile

6M75c = Six-minute activity-75^th^ centile

6M50c = Six-minute activity-50^th^ centile

6M25c = Six-minute activity-25^th^ centile

VMs = Vector magnitudes

Log-linear regression models for 6 minute activity centiles (VMs/6 min segment) versus time since loss of ambulation. Model: $\text{log}\left(\hat{cen}\text{tlie}\right)=A+B\left(\text{year}s\right)$. Key for significance: ns: *P* > 0.05.

* *P* < 0.001.

**TABLE 6.** Association of Six-Minute Activity Centiles and Indexed QMT Metric (N = 97 Participants, n = 168 Wears)

Centile	Total Arm	Total Leg	QMT Total
6M25c	r_s_: 0.307	r_s_: 0.255*	r_s_: 0.289^†^
6M50c	r_s_: 0.505^†^	r_s_: 0.523^†^	r_s_: 0.529^†^
6M75c	r_s_: 0.595^†^	r_s_: 0.603^†^	r_s_: 0.616^†^
6M95c	r_s_: 0.646^†^	r_s_: 0.608^†^	r_s_: 0.647^†^
VMs per minute	r_s_: 0.619^†^	r_s_: 0.615^†^	r_s_: 0.636^†^

Abbreviations:

6M95c = Six-minute activity-95^th^ centile

6M75c = Six-minute activity-75^th^ centile

6M50c = Six-minute activity-50^th^ centile

6M25c = Six-minute activity-25^th^ centile

QMT = Quantitative muscle testing

VMs = Vector magnitudes

Spearman’s rho (r_s_) coefficients used to assess strength of association between 6 MA centiles and four dimensions of indexed QMT (elbow flexion, elbow extension, knee flexion, knee extension). Coefficients less than 0.4 in magnitude considered weak, between 0.4 and 0.7 considered moderately strong, and greater than 0.7 considered strong. Key for significance: ns: *P* > 0.05.

* *P* < 0.01.

† *P* < 0.001.
