# Supplementary material for: Six-Minute Activity-95th Centile, a Novel Wearable-Derived Clinical Outcome Assessment for Duchenne Muscular Dystrophy
Source: Pediatr Neurol. Author manuscript; Available in PMC 2026 Jun 26. (PMC13306447; doi:10.1016/j.pediatrneurol.2025.11.017)
Supplement: 1 [file NIHMS2187307-supplement-1.docx]

**For Appendix

Table A.1. Quantification of indexed QMT scores.

|  | **Median**  **(IQR)** |
| --- | --- |
|  |  |
| *Total Arm QMT (lbs)* | 20.65  (12.00, 30.15) |
| *Indexed Arm QMT (lbs/yr)* | 1.74  (0.80, 2.65) |
| *Total Leg QMT (lbs)* | 32.30  (19.95, 42.35) |
| *Indexed Leg QMT (lbs/yr)* | 2.54  (1.33, 3.83) |
| *Total QMT (lbs)* | 52.45  (32.55, 71.85) |
| *Indexed Total QMT (lbs/yr)* | 4.48  (2.22, 6.67) |

Median and interquartile range of quantitative muscle testing scores. Arm QMT the summed scores of right and left elbow flexion and extension measures. Leg QMT the summed scores of right and left knee flexion and extension measures. Total QMT the sum of total arm and total leg QMT. Indexed QMT calculated by dividing total scores by participant age up to 18 years.
