# Supplementary material for: Six-Minute Activity-95th Centile, a Novel Wearable-Derived Clinical Outcome Assessment for Duchenne Muscular Dystrophy
Source: Pediatr Neurol. Author manuscript; Available in PMC 2026 Jun 26. (PMC13306447; doi:10.1016/j.pediatrneurol.2025.11.017)
Supplement: 2 [file NIHMS2187307-supplement-2.docx]

**For Appendix

Table A.2. Holm corrected p-values for six minute activity correlations with QMT.

|  | Total Arm | Total Leg | QMT Total |
| --- | --- | --- | --- |
| 6M25c | p<0.001 | p=0.002 | P<0.001 |
| 6M50c | p<0.001 | p<0.001 | p<0.001 |
| 6M75c | p<0.001 | p<0.001 | p<0.001 |
| 6M95c | p<0.001 | p<0.001 | p<0.001 |
| VMs Per Minute | p<0.001 | p<0.001 | p<0.001 |

Holm corrected p-values used to assess significance of association between 6MA centiles and dimensions of indexed QMT (total arm, total leg, and composite).
