# Supplementary material for: Six-Minute Activity-95th Centile, a Novel Wearable-Derived Clinical Outcome Assessment for Duchenne Muscular Dystrophy
Source: Pediatr Neurol. Author manuscript; Available in PMC 2026 Jun 26. (PMC13306447; doi:10.1016/j.pediatrneurol.2025.11.017)
Supplement: 3 [file NIHMS2187307-supplement-3.docx]

**For Appendix

Table A.3. Association of six minute activity centiles to cardiac MRI measures. (N=81 participants; n=127 wears)


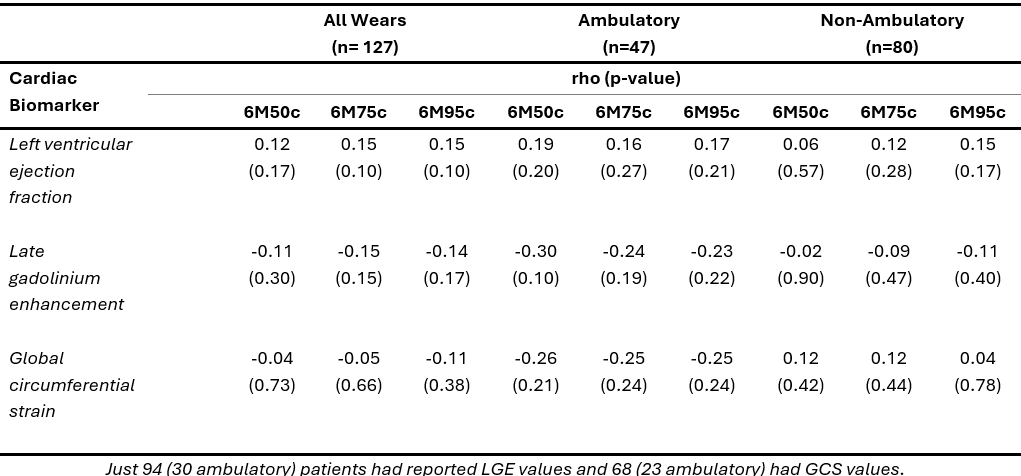


Of the 127 wears, 96 (31 ambulatory) wears had associated LGE values and 69 (24 ambulatory) had global circumferential strain (Ecc) values. A level of p<0.05 is considered significant.
