# Supplementary figures and images for: Six-Minute Activity-95th Centile, a Novel Wearable-Derived Clinical Outcome Assessment for Duchenne Muscular Dystrophy
Source: Pediatr Neurol. Author manuscript; Available in PMC 2026 Jun 26. (PMC13306447; doi:10.1016/j.pediatrneurol.2025.11.017)

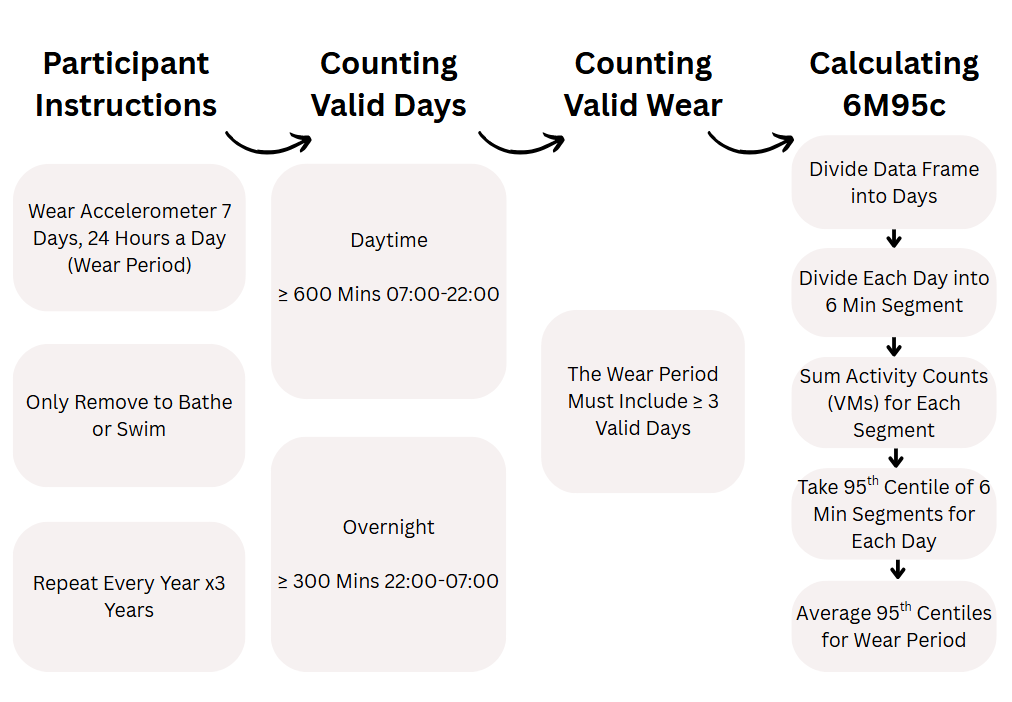


Supplementary Figure 1. Flowchart of accelerometer protocol and calculation of 6M95c.

Supplement: 4 [file NIHMS2187307-supplement-4.docx]
