# Supplementary material for: Six-Minute Activity-95th Centile, a Novel Wearable-Derived Clinical Outcome Assessment for Duchenne Muscular Dystrophy
Source: Pediatr Neurol. Author manuscript; Available in PMC 2026 Jun 26. (PMC13306447; doi:10.1016/j.pediatrneurol.2025.11.017)
Supplement: 5 [file NIHMS2187307-supplement-5.docx]

Supplementary Figure 2. Example of accelerometer placement on dominant wrist.
