# Supplementary material for: Six-Minute Activity-95th Centile, a Novel Wearable-Derived Clinical Outcome Assessment for Duchenne Muscular Dystrophy
Source: Pediatr Neurol. Author manuscript; Available in PMC 2026 Jun 26. (PMC13306447; doi:10.1016/j.pediatrneurol.2025.11.017)

6M95c_analysis

2025-03-04

##Reproducible Code for calculating 6M95c From VMs

##vec <- dframe_day$vm

## apply.sixmin=function(vec,percentile.list=c(0.95),window.size=24)
## {
## #calculate sliding window values
## start.index=1:(length(vec)-window.size+1)
## stop.index=window.size:length(vec)
## values=vector(length=length(start.index))
## for(i in 1:length(start.index))
## {
## values[i]=sum(vec[start.index[i]:stop.index[i]])/window.size
## }
## #calculate percentile based on the values
## out=quantile(values,percentile.list,na.rm=T)
## names(out)=paste("sixmin_",format(round(percentile.list,digits=2)),sep="")
## return(out)
## }


##Mann-Whitney and Wilcoxon
library(readr)
library(sasLM)

## Loading required package: mvtnorm

library(summarytools)
library(corrplot)

## corrplot 0.95 loaded

library(broom)
library(ggplot2)

setwd("//drives.vumc.org/phi/Car/Soslow Research/Joy Actigraphy/Joy Manuscript Info/6MPA Manuscript")

vmperqmt <- read_csv("//drives.vumc.org/phi/Car/Soslow Research/Joy Actigraphy/Joy Manuscript info/6MPA Manuscript/DMDNHSR56Longitudina-ActivityPercentilesQ_DATA_LABELS_2024-07-10_1731.csv")

## Rows: 176 Columns: 55

## ── Column specification ────────────────────────────────────────────────────────
## Delimiter: ","
## chr (5): studyid, Event Name, Date of Birth, Visit Date, yrloa
## dbl (50): age, amb, ageloa, elbflex, elbext, knflex, knext, arm_tot, qmt_ind...
##
## ℹ Use `spec()` to retrieve the full column specification for this data.
## ℹ Specify the column types or set `show_col_types = FALSE` to quiet this message.

descr(vmperqmt$amb_95, stats= "fivenum")

## Descriptive Statistics
## vmperqmt$amb_95
## N: 176
##
## amb_95
## ------------ ---------
## Min 337.91
## Q1 1155.45
## Median 1364.26
## Q3 1601.24
## Max 2779.31

descr(vmperqmt$amb_75, stats= "fivenum")

## Descriptive Statistics
## vmperqmt$amb_75
## N: 176
##
## amb_75
## ------------ ---------
## Min 96.52
## Q1 480.72
## Median 600.83
## Q3 819.13
## Max 1177.48

descr(vmperqmt$amb_50, stats= "fivenum")

## Descriptive Statistics
## vmperqmt$amb_50
## N: 176
##
## amb_50
## ------------ --------
## Min 33.59
## Q1 168.07
## Median 211.75
## Q3 318.87
## Max 608.96

descr(vmperqmt$amb_25, stats= "fivenum")

## Descriptive Statistics
## vmperqmt$amb_25
## N: 176
##
## amb_25
## ------------ --------
## Min 0.31
## Q1 17.49
## Median 24.59
## Q3 36.21
## Max 127.31

descr(vmperqmt$namb_95, stats= "fivenum")

## Descriptive Statistics
## vmperqmt$namb_95
## N: 176
##
## namb_95
## ------------ ---------
## Min 24.42
## Q1 393.79
## Median 665.38
## Q3 889.04
## Max 1618.15

descr(vmperqmt$namb_75, stats= "fivenum")

## Descriptive Statistics
## vmperqmt$namb_75
## N: 176
##
## namb_75
## ------------ ---------
## Min 3.25
## Q1 162.52
## Median 313.42
## Q3 457.82
## Max 1005.02

descr(vmperqmt$namb_50, stats= "fivenum")

## Descriptive Statistics
## vmperqmt$namb_50
## N: 176
##
## namb_50
## ------------ ---------
## Min 0.21
## Q1 56.81
## Median 131.86
## Q3 204.55
## Max 524.31

descr(vmperqmt$namb_25, stats= "fivenum")

## Descriptive Statistics
## vmperqmt$namb_25
## N: 176
##
## namb_25
## ------------ ---------
## Min 0.00
## Q1 3.94
## Median 13.91
## Q3 32.44
## Max 77.39

wilcox.test(vmperqmt$amb_95, vmperqmt$namb_95, paired=FALSE)

##
## Wilcoxon rank sum test with continuity correction
##
## data: vmperqmt$amb_95 and vmperqmt$namb_95
## W = 6538, p-value < 2.2e-16
## alternative hypothesis: true location shift is not equal to 0

wilcox.test(vmperqmt$amb_75, vmperqmt$namb_75, paired=FALSE)

##
## Wilcoxon rank sum test with continuity correction
##
## data: vmperqmt$amb_75 and vmperqmt$namb_75
## W = 6119, p-value = 5.67e-14
## alternative hypothesis: true location shift is not equal to 0

wilcox.test(vmperqmt$amb_50, vmperqmt$namb_50, paired=FALSE)

##
## Wilcoxon rank sum test with continuity correction
##
## data: vmperqmt$amb_50 and vmperqmt$namb_50
## W = 5553, p-value = 7.15e-09
## alternative hypothesis: true location shift is not equal to 0

wilcox.test(vmperqmt$amb_25, vmperqmt$namb_25, paired=FALSE)

##
## Wilcoxon rank sum test with continuity correction
##
## data: vmperqmt$amb_25 and vmperqmt$namb_25
## W = 4821, p-value = 0.0003815
## alternative hypothesis: true location shift is not equal to 0

descr(vmperqmt$pamb_95, stats= "fivenum") #pamb for loa

## Descriptive Statistics
## vmperqmt$pamb_95
## N: 176
##
## pamb_95
## ------------ ---------
## Min 337.91
## Q1 636.68
## Median 1306.66
## Q3 1521.07
## Max 1785.08

descr(vmperqmt$pamb_75, stats= "fivenum")

## Descriptive Statistics
## vmperqmt$pamb_75
## N: 176
##
## pamb_75
## ------------ ---------
## Min 96.52
## Q1 315.74
## Median 553.42
## Q3 882.31
## Max 1019.66

descr(vmperqmt$pamb_50, stats= "fivenum")

## Descriptive Statistics
## vmperqmt$pamb_50
## N: 176
##
## pamb_50
## ------------ ---------
## Min 35.07
## Q1 107.26
## Median 221.07
## Q3 393.58
## Max 528.65

descr(vmperqmt$pamb_25, stats= "fivenum")

## Descriptive Statistics
## vmperqmt$pamb_25
## N: 176
##
## pamb_25
## ------------ ---------
## Min 3.55
## Q1 7.62
## Median 22.23
## Q3 54.07
## Max 60.22

descr(vmperqmt$pnamb_95, stats= "fivenum") #pnamb post-loa

## Descriptive Statistics
## vmperqmt$pnamb_95
## N: 176
##
## pnamb_95
## ------------ ----------
## Min 276.03
## Q1 689.28
## Median 898.75
## Q3 1139.50
## Max 1386.10

descr(vmperqmt$pnamb_75, stats= "fivenum")

## Descriptive Statistics
## vmperqmt$pnamb_75
## N: 176
##
## pnamb_75
## ------------ ----------
## Min 90.85
## Q1 296.54
## Median 453.48
## Q3 558.71
## Max 776.99

descr(vmperqmt$pnamb_50, stats= "fivenum")

## Descriptive Statistics
## vmperqmt$pnamb_50
## N: 176
##
## pnamb_50
## ------------ ----------
## Min 27.50
## Q1 118.46
## Median 195.86
## Q3 238.56
## Max 404.22

descr(vmperqmt$pnamb_25, stats= "fivenum")

## Descriptive Statistics
## vmperqmt$pnamb_25
## N: 176
##
## pnamb_25
## ------------ ----------
## Min 0.00
## Q1 3.94
## Median 23.78
## Q3 36.98
## Max 42.88

wilcox.test(vmperqmt$pamb_95, vmperqmt$pnamb_95, paired=TRUE)

##
## Wilcoxon signed rank exact test
##
## data: vmperqmt$pamb_95 and vmperqmt$pnamb_95
## V = 53, p-value = 0.08301
## alternative hypothesis: true location shift is not equal to 0

wilcox.test(vmperqmt$pamb_75, vmperqmt$pnamb_75, paired=TRUE)

##
## Wilcoxon signed rank exact test
##
## data: vmperqmt$pamb_75 and vmperqmt$pnamb_75
## V = 54, p-value = 0.06738
## alternative hypothesis: true location shift is not equal to 0

wilcox.test(vmperqmt$pamb_50, vmperqmt$pnamb_50, paired=TRUE)

##
## Wilcoxon signed rank exact test
##
## data: vmperqmt$pamb_50 and vmperqmt$pnamb_50
## V = 50, p-value = 0.1475
## alternative hypothesis: true location shift is not equal to 0

wilcox.test(vmperqmt$pamb_25, vmperqmt$pnamb_25, paired=TRUE)

##
## Wilcoxon signed rank exact test
##
## data: vmperqmt$pamb_25 and vmperqmt$pnamb_25
## V = 50, p-value = 0.1475
## alternative hypothesis: true location shift is not equal to 0

wilcox.test(vmperqmt$visit1_95, vmperqmt$visit3_95, paired=TRUE)

##
## Wilcoxon signed rank exact test
##
## data: vmperqmt$visit1_95 and vmperqmt$visit3_95
## V = 358, p-value = 5.528e-06
## alternative hypothesis: true location shift is not equal to 0

wilcox.test(vmperqmt$visit1_75, vmperqmt$visit3_75, paired=TRUE)

##
## Wilcoxon signed rank exact test
##
## data: vmperqmt$visit1_75 and vmperqmt$visit3_75
## V = 340, p-value = 9.596e-05
## alternative hypothesis: true location shift is not equal to 0

wilcox.test(vmperqmt$visit1_50, vmperqmt$visit3_50, paired=TRUE)

##
## Wilcoxon signed rank exact test
##
## data: vmperqmt$visit1_50 and vmperqmt$visit3_50
## V = 317, p-value = 0.001385
## alternative hypothesis: true location shift is not equal to 0

wilcox.test(vmperqmt$visit1_25, vmperqmt$visit3_25, paired=TRUE)

## Warning in wilcox.test.default(vmperqmt$visit1_25, vmperqmt$visit3_25, paired =
## TRUE): cannot compute exact p-value with zeroes

##
## Wilcoxon signed rank test with continuity correction
##
## data: vmperqmt$visit1_25 and vmperqmt$visit3_25
## V = 216, p-value = 0.3097
## alternative hypothesis: true location shift is not equal to 0

descr(vmperqmt$visit1_95, stats= "fivenum")

## Descriptive Statistics
## vmperqmt$visit1_95
## N: 176
##
## visit1_95
## ------------ -----------
## Min 78.88
## Q1 545.84
## Median 834.60
## Q3 1340.16
## Max 1785.08

descr(vmperqmt$visit1_75, stats= "fivenum")

## Descriptive Statistics
## vmperqmt$visit1_75
## N: 176
##
## visit1_75
## ------------ -----------
## Min 5.88
## Q1 245.65
## Median 406.94
## Q3 622.12
## Max 1143.63

descr(vmperqmt$visit1_50, stats= "fivenum")

## Descriptive Statistics
## vmperqmt$visit1_50
## N: 176
##
## visit1_50
## ------------ -----------
## Min 0.58
## Q1 99.32
## Median 169.30
## Q3 235.36
## Max 608.96

descr(vmperqmt$visit1_25, stats= "fivenum")

## Descriptive Statistics
## vmperqmt$visit1_25
## N: 176
##
## visit1_25
## ------------ -----------
## Min 0.00
## Q1 7.62
## Median 28.43
## Q3 37.33
## Max 54.07

descr(vmperqmt$visit3_95, stats= "fivenum")

## Descriptive Statistics
## vmperqmt$visit3_95
## N: 176
##
## visit3_95
## ------------ -----------
## Min 28.32
## Q1 339.13
## Median 712.08
## Q3 980.47
## Max 1386.10

descr(vmperqmt$visit3_75, stats= "fivenum")

## Descriptive Statistics
## vmperqmt$visit3_75
## N: 176
##
## visit3_75
## ------------ -----------
## Min 5.02
## Q1 206.38
## Median 349.08
## Q3 464.39
## Max 776.99

descr(vmperqmt$visit3_50, stats= "fivenum")

## Descriptive Statistics
## vmperqmt$visit3_50
## N: 176
##
## visit3_50
## ------------ -----------
## Min 0.21
## Q1 73.83
## Median 147.90
## Q3 219.55
## Max 404.22

descr(vmperqmt$visit3_25, stats= "fivenum")

## Descriptive Statistics
## vmperqmt$visit3_25
## N: 176
##
## visit3_25
## ------------ -----------
## Min 0.00
## Q1 3.94
## Median 18.64
## Q3 36.98
## Max 63.50

##QMT Correlations

descr(vmperqmt$arm_tot, stats= "fivenum")

## Descriptive Statistics
## vmperqmt$arm_tot
## N: 176
##
## arm_tot
## ------------ ---------
## Min 0.00
## Q1 11.90
## Median 20.60
## Q3 29.80
## Max 132.00

descr(vmperqmt$qmt_ind_arm, stats= "fivenum")

## Descriptive Statistics
## vmperqmt$qmt_ind_arm
## N: 176
##
## qmt_ind_arm
## ------------ -------------
## Min 0.00
## Q1 0.79
## Median 1.73
## Q3 2.69
## Max 9.00

descr(vmperqmt$leg_tot, stats= "fivenum")

## Descriptive Statistics
## vmperqmt$leg_tot
## N: 176
##
## leg_tot
## ------------ ---------
## Min 0.00
## Q1 20.00
## Median 31.60
## Q3 42.30
## Max 155.00

descr(vmperqmt$qmt_ind_leg, stats= "fivenum")

## Descriptive Statistics
## vmperqmt$qmt_ind_leg
## N: 176
##
## qmt_ind_leg
## ------------ -------------
## Min 0.00
## Q1 1.29
## Median 2.51
## Q3 3.85
## Max 17.22

descr(vmperqmt$qmt_tot, stats= "fivenum")

## Descriptive Statistics
## vmperqmt$qmt_tot
## N: 176
##
## qmt_tot
## ------------ ---------
## Min 6.30
## Q1 32.80
## Median 52.10
## Q3 71.80
## Max 283.00

descr(vmperqmt$qmt_ind_tot, stats= "fivenum")

## Descriptive Statistics
## vmperqmt$qmt_ind_tot
## N: 176
##
## qmt_ind_tot
## ------------ -------------
## Min 0.35
## Q1 2.21
## Median 4.47
## Q3 6.68
## Max 25.11

cor.test(vmperqmt$qmt_ind_arm, vmperqmt$all_95, method = "spearman", exact=FALSE)

##
## Spearman's rank correlation rho
##
## data: vmperqmt$qmt_ind_arm and vmperqmt$all_95
## S = 240074, p-value < 2.2e-16
## alternative hypothesis: true rho is not equal to 0
## sample estimates:
## rho
## 0.6548275

pValarm1 <- cor.test(vmperqmt$qmt_ind_arm, vmperqmt$all_95, method = "spearman", exact=FALSE)$p.value
cor.test(vmperqmt$qmt_ind_leg, vmperqmt$all_95, method = "spearman", exact=FALSE)

##
## Spearman's rank correlation rho
##
## data: vmperqmt$qmt_ind_leg and vmperqmt$all_95
## S = 261554, p-value < 2.2e-16
## alternative hypothesis: true rho is not equal to 0
## sample estimates:
## rho
## 0.623944

pValleg1 <- cor.test(vmperqmt$qmt_ind_leg, vmperqmt$all_95, method = "spearman", exact=FALSE)$p.value
cor.test(vmperqmt$qmt_ind_tot, vmperqmt$all_95, method = "spearman", exact=FALSE)

##
## Spearman's rank correlation rho
##
## data: vmperqmt$qmt_ind_tot and vmperqmt$all_95
## S = 237061, p-value < 2.2e-16
## alternative hypothesis: true rho is not equal to 0
## sample estimates:
## rho
## 0.6591605

pValtot1 <- cor.test(vmperqmt$qmt_ind_tot, vmperqmt$all_95, method = "spearman", exact=FALSE)$p.value

cor.test(vmperqmt$qmt_ind_arm, vmperqmt$all_75, method = "spearman", exact=FALSE)

##
## Spearman's rank correlation rho
##
## data: vmperqmt$qmt_ind_arm and vmperqmt$all_75
## S = 271917, p-value < 2.2e-16
## alternative hypothesis: true rho is not equal to 0
## sample estimates:
## rho
## 0.6090443

pValarm2 <- cor.test(vmperqmt$qmt_ind_arm, vmperqmt$all_75, method = "spearman", exact=FALSE)$p.value
cor.test(vmperqmt$qmt_ind_leg, vmperqmt$all_75, method = "spearman", exact=FALSE)

##
## Spearman's rank correlation rho
##
## data: vmperqmt$qmt_ind_leg and vmperqmt$all_75
## S = 260927, p-value < 2.2e-16
## alternative hypothesis: true rho is not equal to 0
## sample estimates:
## rho
## 0.6248455

pValleg2 <- cor.test(vmperqmt$qmt_ind_leg, vmperqmt$all_75, method = "spearman", exact=FALSE)$p.value
cor.test(vmperqmt$qmt_ind_tot, vmperqmt$all_75, method = "spearman", exact=FALSE)

##
## Spearman's rank correlation rho
##
## data: vmperqmt$qmt_ind_tot and vmperqmt$all_75
## S = 255668, p-value < 2.2e-16
## alternative hypothesis: true rho is not equal to 0
## sample estimates:
## rho
## 0.6324079

pValtot2 <- cor.test(vmperqmt$qmt_ind_tot, vmperqmt$all_75, method = "spearman", exact=FALSE)$p.value

cor.test(vmperqmt$qmt_ind_arm, vmperqmt$all_50, method = "spearman", exact=FALSE)

##
## Spearman's rank correlation rho
##
## data: vmperqmt$qmt_ind_arm and vmperqmt$all_50
## S = 336802, p-value = 2.533e-12
## alternative hypothesis: true rho is not equal to 0
## sample estimates:
## rho
## 0.5157555

pValarm3 <- cor.test(vmperqmt$qmt_ind_arm, vmperqmt$all_50, method = "spearman", exact=FALSE)$p.value
cor.test(vmperqmt$qmt_ind_leg, vmperqmt$all_50, method = "spearman", exact=FALSE)

##
## Spearman's rank correlation rho
##
## data: vmperqmt$qmt_ind_leg and vmperqmt$all_50
## S = 321119, p-value = 1.775e-13
## alternative hypothesis: true rho is not equal to 0
## sample estimates:
## rho
## 0.5383042

pValleg3 <- cor.test(vmperqmt$qmt_ind_leg, vmperqmt$all_50, method = "spearman", exact=FALSE)$p.value
cor.test(vmperqmt$qmt_ind_tot, vmperqmt$all_50, method = "spearman", exact=FALSE)

##
## Spearman's rank correlation rho
##
## data: vmperqmt$qmt_ind_tot and vmperqmt$all_50
## S = 320100, p-value = 1.484e-13
## alternative hypothesis: true rho is not equal to 0
## sample estimates:
## rho
## 0.5397692

pValtot3 <- cor.test(vmperqmt$qmt_ind_tot, vmperqmt$all_50, method = "spearman", exact=FALSE)$p.value

cor.test(vmperqmt$qmt_ind_arm, vmperqmt$all_25, method = "spearman", exact=FALSE)

##
## Spearman's rank correlation rho
##
## data: vmperqmt$qmt_ind_arm and vmperqmt$all_25
## S = 486847, p-value = 0.0001103
## alternative hypothesis: true rho is not equal to 0
## sample estimates:
## rho
## 0.300025

pValarm4 <- cor.test(vmperqmt$qmt_ind_arm, vmperqmt$all_25, method = "spearman", exact=FALSE)$p.value
cor.test(vmperqmt$qmt_ind_leg, vmperqmt$all_25, method = "spearman", exact=FALSE)

##
## Spearman's rank correlation rho
##
## data: vmperqmt$qmt_ind_leg and vmperqmt$all_25
## S = 526660, p-value = 0.001915
## alternative hypothesis: true rho is not equal to 0
## sample estimates:
## rho
## 0.2427826

pValleg4 <- cor.test(vmperqmt$qmt_ind_leg, vmperqmt$all_25, method = "spearman", exact=FALSE)$p.value
cor.test(vmperqmt$qmt_ind_tot, vmperqmt$all_25, method = "spearman", exact=FALSE)

##
## Spearman's rank correlation rho
##
## data: vmperqmt$qmt_ind_tot and vmperqmt$all_25
## S = 503168, p-value = 0.0003833
## alternative hypothesis: true rho is not equal to 0
## sample estimates:
## rho
## 0.2765586

pValtot4 <- cor.test(vmperqmt$qmt_ind_tot, vmperqmt$all_25, method = "spearman", exact=FALSE)$p.value

cor.test(vmperqmt$qmt_ind_arm, vmperqmt$vmtot, method = "spearman", exact=FALSE)

##
## Spearman's rank correlation rho
##
## data: vmperqmt$qmt_ind_arm and vmperqmt$vmtot
## S = 279091, p-value < 2.2e-16
## alternative hypothesis: true rho is not equal to 0
## sample estimates:
## rho
## 0.5987312

pValarm5 <- cor.test(vmperqmt$qmt_ind_arm, vmperqmt$vmtot, method = "spearman", exact=FALSE)$p.value
cor.test(vmperqmt$qmt_ind_leg, vmperqmt$vmtot, method = "spearman", exact=FALSE)

##
## Spearman's rank correlation rho
##
## data: vmperqmt$qmt_ind_leg and vmperqmt$vmtot
## S = 281376, p-value < 2.2e-16
## alternative hypothesis: true rho is not equal to 0
## sample estimates:
## rho
## 0.5954458

pValleg5 <- cor.test(vmperqmt$qmt_ind_leg, vmperqmt$vmtot, method = "spearman", exact=FALSE)$p.value
cor.test(vmperqmt$qmt_ind_tot, vmperqmt$vmtot, method = "spearman", exact=FALSE)

##
## Spearman's rank correlation rho
##
## data: vmperqmt$qmt_ind_tot and vmperqmt$vmtot
## S = 268973, p-value < 2.2e-16
## alternative hypothesis: true rho is not equal to 0
## sample estimates:
## rho
## 0.6132783

pValtot5 <- cor.test(vmperqmt$qmt_ind_tot, vmperqmt$vmtot, method = "spearman", exact=FALSE)$p.value

cor.test(vmperqmt$qmt_ind_arm, vmperqmt$vmpermin, method = "spearman", exact=FALSE)

##
## Spearman's rank correlation rho
##
## data: vmperqmt$qmt_ind_arm and vmperqmt$vmpermin
## S = 257829, p-value < 2.2e-16
## alternative hypothesis: true rho is not equal to 0
## sample estimates:
## rho
## 0.6292998

pValarm6 <- cor.test(vmperqmt$qmt_ind_arm, vmperqmt$vmpermin, method = "spearman", exact=FALSE)$p.value
cor.test(vmperqmt$qmt_ind_leg, vmperqmt$vmpermin, method = "spearman", exact=FALSE)

##
## Spearman's rank correlation rho
##
## data: vmperqmt$qmt_ind_leg and vmperqmt$vmpermin
## S = 259501, p-value < 2.2e-16
## alternative hypothesis: true rho is not equal to 0
## sample estimates:
## rho
## 0.6268958

pValleg6 <- cor.test(vmperqmt$qmt_ind_leg, vmperqmt$vmpermin, method = "spearman", exact=FALSE)$p.value
cor.test(vmperqmt$qmt_ind_tot, vmperqmt$vmpermin, method = "spearman", exact=FALSE)

##
## Spearman's rank correlation rho
##
## data: vmperqmt$qmt_ind_tot and vmperqmt$vmpermin
## S = 246470, p-value < 2.2e-16
## alternative hypothesis: true rho is not equal to 0
## sample estimates:
## rho
## 0.6456325

pValtot6 <- cor.test(vmperqmt$qmt_ind_tot, vmperqmt$vmpermin, method = "spearman", exact=FALSE)$p.value

cor.test(vmperqmt$qmt_ind_arm, vmperqmt$vmperday, method = "spearman", exact=FALSE)

##
## Spearman's rank correlation rho
##
## data: vmperqmt$qmt_ind_arm and vmperqmt$vmperday
## S = 256417, p-value < 2.2e-16
## alternative hypothesis: true rho is not equal to 0
## sample estimates:
## rho
## 0.6313299

pValarm7 <- cor.test(vmperqmt$qmt_ind_arm, vmperqmt$vmperday, method = "spearman", exact=FALSE)$p.value
cor.test(vmperqmt$qmt_ind_leg, vmperqmt$vmperday, method = "spearman", exact=FALSE)

##
## Spearman's rank correlation rho
##
## data: vmperqmt$qmt_ind_leg and vmperqmt$vmperday
## S = 257694, p-value < 2.2e-16
## alternative hypothesis: true rho is not equal to 0
## sample estimates:
## rho
## 0.6294939

pValleg7 <- cor.test(vmperqmt$qmt_ind_leg, vmperqmt$vmperday, method = "spearman", exact=FALSE)$p.value
cor.test(vmperqmt$qmt_ind_tot, vmperqmt$vmperday, method = "spearman", exact=FALSE)

##
## Spearman's rank correlation rho
##
## data: vmperqmt$qmt_ind_tot and vmperqmt$vmperday
## S = 245043, p-value < 2.2e-16
## alternative hypothesis: true rho is not equal to 0
## sample estimates:
## rho
## 0.6476842

pValtot7 <- cor.test(vmperqmt$qmt_ind_tot, vmperqmt$vmperday, method = "spearman", exact=FALSE)$p.value

pQMT <- c(pValarm1, pValleg1, pValtot1, pValarm2, pValleg2, pValtot2, pValarm3, pValleg3, pValtot3, pValarm4, pValleg4, pValtot4, pValarm5, pValleg5, pValtot5, pValarm6, pValleg6, pValtot6, pValarm7, pValleg7, pValtot7)
p.adjust(pQMT, method="holm")

## [1] 8.844152e-20 1.036674e-17 4.160902e-20 9.161867e-17 9.752532e-18
## [6] 3.913326e-18 1.013169e-11 8.901396e-13 8.901396e-13 3.309657e-04
## [11] 1.914881e-03 7.666244e-04 3.935317e-16 5.620870e-16 5.243355e-17
## [16] 5.637244e-18 7.530600e-18 4.189814e-19 4.418028e-18 5.637244e-18
## [21] 3.068144e-19

##Participant Demographic Comparison
library(readr)
library(sasLM)
library(summarytools)
library(corrplot)
library(broom)
library(ggplot2)

#t tests of vm percentiles total pop vs amb

partinfo <- read_csv("//drives.vumc.org/phi/Car/Soslow Research/Joy Actigraphy/Joy Manuscript info/6MPA Manuscript/PartInfo.csv")

## Rows: 139 Columns: 22
## ── Column specification ────────────────────────────────────────────────────────
## Delimiter: ","
## chr (5): SubjectID, Amb, Included, ambinc, ambout
## dbl (17): sex, age, ht, wt, BMIall, age_in_months, height_cm, weight_kg, bmi...
##
## ℹ Use `spec()` to retrieve the full column specification for this data.
## ℹ Specify the column types or set `show_col_types = FALSE` to quiet this message.

sink("outputfilepartinfo.txt")


descr(partinfo$ageinc, stats= "fivenum")
descr(partinfo$htinc, stats= "fivenum")
descr(partinfo$wtinc, stats= "fivenum")
descr(partinfo$bmiinc, stats= "fivenum")
descr(partinfo$ageout, stats= "fivenum")
descr(partinfo$htout, stats= "fivenum")
descr(partinfo$wtout, stats= "fivenum")
descr(partinfo$bmiout, stats= "fivenum")


wilcox.test(partinfo$ageinc, partinfo$ageout, paired=FALSE)
wilcox.test(partinfo$htinc, partinfo$htout, paired=FALSE)
wilcox.test(partinfo$wtinc, partinfo$wtout, paired=FALSE)
wilcox.test(partinfo$bmiinc, partinfo$bmiout, paired=FALSE)


Input =(
 "Included Ambulatory NonAmbulatory
 Yes 47 53
 No 18 21
 ")
myMatrix = as.matrix(read.table(textConnection(Input),
 header=TRUE,
 row.names=1))
myMatrix

chisq.test(myMatrix, correct=FALSE)

sink()

##Predictive Curves

vmperqmt <- read_csv("//drives.vumc.org/phi/Car/Soslow Research/Joy Actigraphy/Joy Manuscript info/6MPA Manuscript/Pred Model.csv")

## New names:
## Rows: 116 Columns: 20
## ── Column specification
## ──────────────────────────────────────────────────────── Delimiter: "," chr
## (1): subjectid dbl (18): yrsaftloa, loaper25, loaper50, loaper75, loaper95,
## vmtot, vmpermin... lgl (1): ...10
## ℹ Use `spec()` to retrieve the full column specification for this data. ℹ
## Specify the column types or set `show_col_types = FALSE` to quiet this message.
## • `` -> `...10`
## • `95zero` -> `95zero...11`
## • `95one` -> `95one...12`
## • `95zero` -> `95zero...14`
## • `95one` -> `95one...15`
## • `95two` -> `95two...16`
## • `95two` -> `95two...17`
## • `95three` -> `95three...18`
## • `95three` -> `95three...19`

library(lme4)

## Loading required package: Matrix

model1=lmer(log(loaper95)~yrsaftloa+(1|subjectid),
 data=vmperqmt)
summary(model1)

## Linear mixed model fit by REML ['lmerMod']
## Formula: log(loaper95) ~ yrsaftloa + (1 | subjectid)
## Data: vmperqmt
##
## REML criterion at convergence: 182.9
##
## Scaled residuals:
## Min 1Q Median 3Q Max
## -2.73604 -0.32517 0.08496 0.41009 2.07371
##
## Random effects:
## Groups Name Variance Std.Dev.
## subjectid (Intercept) 0.2648 0.5146
## Residual 0.1073 0.3276
## Number of obs: 116, groups: subjectid, 65
##
## Fixed effects:
## Estimate Std. Error t value
## (Intercept) 6.90280 0.09974 69.207
## yrsaftloa -0.17880 0.02006 -8.913
##
## Correlation of Fixed Effects:
## (Intr)
## yrsaftloa -0.693

model2=lmer(log(loaper75)~yrsaftloa+(1|subjectid),
 data=vmperqmt)
summary(model2)

## Linear mixed model fit by REML ['lmerMod']
## Formula: log(loaper75) ~ yrsaftloa + (1 | subjectid)
## Data: vmperqmt
##
## REML criterion at convergence: 244
##
## Scaled residuals:
## Min 1Q Median 3Q Max
## -3.4794 -0.2842 0.0369 0.3605 2.7067
##
## Random effects:
## Groups Name Variance Std.Dev.
## subjectid (Intercept) 0.5385 0.7338
## Residual 0.1591 0.3989
## Number of obs: 116, groups: subjectid, 65
##
## Fixed effects:
## Estimate Std. Error t value
## (Intercept) 6.27633 0.13643 46.006
## yrsaftloa -0.23086 0.02696 -8.565
##
## Correlation of Fixed Effects:
## (Intr)
## yrsaftloa -0.683

model3=lmer(log(loaper50)~yrsaftloa+(1|subjectid),
 data=vmperqmt)
summary(model3)

## Linear mixed model fit by REML ['lmerMod']
## Formula: log(loaper50) ~ yrsaftloa + (1 | subjectid)
## Data: vmperqmt
##
## REML criterion at convergence: 276.5
##
## Scaled residuals:
## Min 1Q Median 3Q Max
## -3.3802 -0.3402 0.0489 0.3886 2.5735
##
## Random effects:
## Groups Name Variance Std.Dev.
## subjectid (Intercept) 0.6972 0.8350
## Residual 0.2165 0.4653
## Number of obs: 116, groups: subjectid, 65
##
## Fixed effects:
## Estimate Std. Error t value
## (Intercept) 5.41290 0.15621 34.65
## yrsaftloa -0.24986 0.03096 -8.07
##
## Correlation of Fixed Effects:
## (Intr)
## yrsaftloa -0.685

model4=lmer(log(loaper25)~yrsaftloa+(1|subjectid),
 data=vmperqmt)
summary(model4)

## Linear mixed model fit by REML ['lmerMod']
## Formula: log(loaper25) ~ yrsaftloa + (1 | subjectid)
## Data: vmperqmt
##
## REML criterion at convergence: 332.7
##
## Scaled residuals:
## Min 1Q Median 3Q Max
## -2.3960 -0.5067 0.1084 0.5320 2.0858
##
## Random effects:
## Groups Name Variance Std.Dev.
## subjectid (Intercept) 0.8645 0.9298
## Residual 0.4403 0.6636
## Number of obs: 116, groups: subjectid, 65
##
## Fixed effects:
## Estimate Std. Error t value
## (Intercept) 3.04221 0.18620 16.338
## yrsaftloa -0.17859 0.03786 -4.718
##
## Correlation of Fixed Effects:
## (Intr)
## yrsaftloa -0.699

model5=lmer(log(vmtot)~yrsaftloa+(1|subjectid),
 data=vmperqmt)
summary(model5)

## Linear mixed model fit by REML ['lmerMod']
## Formula: log(vmtot) ~ yrsaftloa + (1 | subjectid)
## Data: vmperqmt
##
## REML criterion at convergence: 277.9
##
## Scaled residuals:
## Min 1Q Median 3Q Max
## -3.8045 -0.3336 0.0649 0.4220 1.9870
##
## Random effects:
## Groups Name Variance Std.Dev.
## subjectid (Intercept) 0.5185 0.7201
## Residual 0.2782 0.5275
## Number of obs: 116, groups: subjectid, 65
##
## Fixed effects:
## Estimate Std. Error t value
## (Intercept) 16.29289 0.14534 112.10
## yrsaftloa -0.23840 0.02961 -8.05
##
## Correlation of Fixed Effects:
## (Intr)
## yrsaftloa -0.700

model6=lmer(log(vmpermin)~yrsaftloa+(1|subjectid),
 data=vmperqmt)
summary(model6)

## Linear mixed model fit by REML ['lmerMod']
## Formula: log(vmpermin) ~ yrsaftloa + (1 | subjectid)
## Data: vmperqmt
##
## REML criterion at convergence: 213.2
##
## Scaled residuals:
## Min 1Q Median 3Q Max
## -2.66199 -0.30179 0.06799 0.44240 1.92235
##
## Random effects:
## Groups Name Variance Std.Dev.
## subjectid (Intercept) 0.3778 0.6146
## Residual 0.1303 0.3610
## Number of obs: 116, groups: subjectid, 65
##
## Fixed effects:
## Estimate Std. Error t value
## (Intercept) 7.11788 0.11657 61.060
## yrsaftloa -0.19499 0.02325 -8.388
##
## Correlation of Fixed Effects:
## (Intr)
## yrsaftloa -0.689

model7=lmer(log(vmperday)~yrsaftloa+(1|subjectid),
 data=vmperqmt)
summary(model7)

## Linear mixed model fit by REML ['lmerMod']
## Formula: log(vmperday) ~ yrsaftloa + (1 | subjectid)
## Data: vmperqmt
##
## REML criterion at convergence: 222.5
##
## Scaled residuals:
## Min 1Q Median 3Q Max
## -2.87536 -0.31071 0.04598 0.42681 1.84339
##
## Random effects:
## Groups Name Variance Std.Dev.
## subjectid (Intercept) 0.4058 0.6370
## Residual 0.1427 0.3777
## Number of obs: 116, groups: subjectid, 65
##
## Fixed effects:
## Estimate Std. Error t value
## (Intercept) 14.37551 0.12112 118.685
## yrsaftloa -0.20402 0.02418 -8.438
##
## Correlation of Fixed Effects:
## (Intr)
## yrsaftloa -0.689

###Model 6M95c
library(cowplot)
library(sjPlot)

## Learn more about sjPlot with 'browseVignettes("sjPlot")'.
##
## Attaching package: 'sjPlot'
##
## The following objects are masked from 'package:cowplot':
##
## plot_grid, save_plot

library(effects)

## Loading required package: carData
## lattice theme set by effectsTheme()
## See ?effectsTheme for details.

sjPlot::tab_model(model1,
 show.re.var= TRUE,
 pred.labels =c("(Intercept)", "Years After Loss of Ambulation"),
 dv.labels= "Effects of Time on 6M95c")

Effects of Time on 6M95c

Predictors

Estimates

CI

p

(Intercept)

6.90

6.71 – 7.10

<0.001

Years After Loss of Ambulation

-0.18

-0.22 – -0.14

<0.001

Random Effects

σ2

0.11

τ00 subjectid

0.26

ICC

0.71

N subjectid

65

Observations

116

Marginal R2 / Conditional R2

0.473 / 0.848

effects_yrsaftloa1 <- effects::effect(term= "yrsaftloa", mod=model1)
summary(effects_yrsaftloa1)

##
## yrsaftloa effect
## yrsaftloa
## -2 2 6 9 10
## 7.260401 6.545200 5.829999 5.293599 5.114799
##
## Lower 95 Percent Confidence Limits
## yrsaftloa
## -2 2 6 9 10
## 7.001303 6.391625 5.655126 5.030930 4.817953
##
## Upper 95 Percent Confidence Limits
## yrsaftloa
## -2 2 6 9 10
## 7.519498 6.698775 6.004872 5.556268 5.411644

x_yrsaft1 <- as.data.frame(effects_yrsaftloa1)

model1_plot <- ggplot() +
 geom_point(data=vmperqmt, aes(yrsaftloa, log(loaper95)))+
 geom_point(data=x_yrsaft1, aes(x=yrsaftloa, y=fit), color="black")+
 geom_line(data=x_yrsaft1, aes(x=yrsaftloa, y=fit), color="black")+
 geom_ribbon(data= x_yrsaft1, aes(x=yrsaftloa, ymin=lower, ymax=upper), alpha=0.4, fill="gray")+
 labs(x=" Years After LOA", y="6M95c", title="Predictive 95th Centile") + theme_bw()

p1 <-model1_plot


###Model 6M75c

sjPlot::tab_model(model2,
 show.re.var= TRUE,
 pred.labels =c("(Intercept)", "Years After Loss of Ambulation"),
 dv.labels= "Effects of Time on 6M75c")

Effects of Time on 6M75c

Predictors

Estimates

CI

p

(Intercept)

6.28

6.01 – 6.55

<0.001

Years After Loss of Ambulation

-0.23

-0.28 – -0.18

<0.001

Random Effects

σ2

0.16

τ00 subjectid

0.54

ICC

0.77

N subjectid

65

Observations

116

Marginal R2 / Conditional R2

0.444 / 0.873

effects_yrsaftloa2 <- effects::effect(term= "yrsaftloa", mod=model2)
summary(effects_yrsaftloa2)

##
## yrsaftloa effect
## yrsaftloa
## -2 2 6 9 10
## 6.738052 5.814598 4.891144 4.198553 3.967690
##
## Lower 95 Percent Confidence Limits
## yrsaftloa
## -2 2 6 9 10
## 6.386072 5.602466 4.651652 3.842879 3.566492
##
## Upper 95 Percent Confidence Limits
## yrsaftloa
## -2 2 6 9 10
## 7.090033 6.026730 5.130636 4.554228 4.368887

x_yrsaft2 <- as.data.frame(effects_yrsaftloa2)

model2_plot <- ggplot() +
 geom_point(data=vmperqmt, aes(yrsaftloa, log(loaper75)))+
 geom_point(data=x_yrsaft2, aes(x=yrsaftloa, y=fit), color="black")+
 geom_line(data=x_yrsaft2, aes(x=yrsaftloa, y=fit), color="black")+
 geom_ribbon(data= x_yrsaft2, aes(x=yrsaftloa, ymin=lower, ymax=upper), alpha=0.4, fill="gray")+
 labs(x=" Years After LOA", y="6M75c", title="Predictive 75th Centile")+ theme_bw()

p2 <-model2_plot


###Model 6M50c


sjPlot::tab_model(model3,
 show.re.var= TRUE,
 pred.labels =c("(Intercept)", "Years After Loss of Ambulation"),
 dv.labels= "Effects of Time on 6M50c")

Effects of Time on 6M50c

Predictors

Estimates

CI

p

(Intercept)

5.41

5.10 – 5.72

<0.001

Years After Loss of Ambulation

-0.25

-0.31 – -0.19

<0.001

Random Effects

σ2

0.22

τ00 subjectid

0.70

ICC

0.76

N subjectid

65

Observations

116

Marginal R2 / Conditional R2

0.416 / 0.862

effects_yrsaftloa3 <- effects::effect(term= "yrsaftloa", mod=model3)
summary(effects_yrsaftloa3)

##
## yrsaftloa effect
## yrsaftloa
## -2 2 6 9 10
## 5.912612 4.913182 3.913752 3.164179 2.914322
##
## Lower 95 Percent Confidence Limits
## yrsaftloa
## -2 2 6 9 10
## 5.509103 4.670698 3.639615 2.756245 2.454021
##
## Upper 95 Percent Confidence Limits
## yrsaftloa
## -2 2 6 9 10
## 6.316122 5.155666 4.187888 3.572113 3.374622

x_yrsaft3 <- as.data.frame(effects_yrsaftloa3)

model3_plot <- ggplot() +
 geom_point(data=vmperqmt, aes(yrsaftloa, log(loaper50)))+
 geom_point(data=x_yrsaft3, aes(x=yrsaftloa, y=fit), color="black")+
 geom_line(data=x_yrsaft3, aes(x=yrsaftloa, y=fit), color="black")+
 geom_ribbon(data= x_yrsaft3, aes(x=yrsaftloa, ymin=lower, ymax=upper), alpha=0.4, fill="gray")+
 labs(x=" Years After LOA", y="6M50c", title= "Predictive 50th Centile")+ theme_bw()

p3 <-model3_plot


###Model 6M25c


sjPlot::tab_model(model4,
 show.re.var= TRUE,
 pred.labels =c("(Intercept)", "Years After Loss of Ambulation"),
 dv.labels= "Effects of Time on 6M25c")

Effects of Time on 6M25c

Predictors

Estimates

CI

p

(Intercept)

3.04

2.67 – 3.41

<0.001

Years After Loss of Ambulation

-0.18

-0.25 – -0.10

<0.001

Random Effects

σ2

0.44

τ00 subjectid

0.86

ICC

0.66

N subjectid

65

Observations

116

Marginal R2 / Conditional R2

0.203 / 0.731

effects_yrsaftloa4 <- effects::effect(term= "yrsaftloa", mod=model4)
summary(effects_yrsaftloa4)

##
## yrsaftloa effect
## yrsaftloa
## -2 2 6 9 10
## 3.399386 2.685025 1.970664 1.434893 1.256302
##
## Lower 95 Percent Confidence Limits
## yrsaftloa
## -2 2 6 9 10
## 2.9137436 2.3999564 1.6441103 0.9411679 0.6977615
##
## Upper 95 Percent Confidence Limits
## yrsaftloa
## -2 2 6 9 10
## 3.885029 2.970094 2.297217 1.928617 1.814843

x_yrsaft4 <- as.data.frame(effects_yrsaftloa4)

model4_plot <- ggplot() +
 geom_point(data=vmperqmt, aes(yrsaftloa, log(loaper25)))+
 geom_point(data=x_yrsaft4, aes(x=yrsaftloa, y=fit), color="black")+
 geom_line(data=x_yrsaft4, aes(x=yrsaftloa, y=fit), color="black")+
 geom_ribbon(data= x_yrsaft4, aes(x=yrsaftloa, ymin=lower, ymax=upper), alpha=0.4, fill="gray")+
 labs(x=" Years After LOA", y="6M25c", title="Predictive 25th Centile") + theme_bw()
p4 <-model4_plot


###Model VMTot


sjPlot::tab_model(model5,
 show.re.var= TRUE,
 pred.labels =c("(Intercept)", "Years After Loss of Ambulation"),
 dv.labels= "Effects of Time on Total VMs")

Effects of Time on Total VMs

Predictors

Estimates

CI

p

(Intercept)

16.29

16.00 – 16.58

<0.001

Years After Loss of Ambulation

-0.24

-0.30 – -0.18

<0.001

Random Effects

σ2

0.28

τ00 subjectid

0.52

ICC

0.65

N subjectid

65

Observations

116

Marginal R2 / Conditional R2

0.427 / 0.800

effects_yrsaftloa5 <- effects::effect(term= "yrsaftloa", mod=model5)
summary(effects_yrsaftloa5)

##
## yrsaftloa effect
## yrsaftloa
## -2 2 6 9 10
## 16.76969 15.81609 14.86250 14.14731 13.90891
##
## Lower 95 Percent Confidence Limits
## yrsaftloa
## -2 2 6 9 10
## 16.39030 15.59384 14.60755 13.76134 13.47218
##
## Upper 95 Percent Confidence Limits
## yrsaftloa
## -2 2 6 9 10
## 17.14907 16.03835 15.11745 14.53328 14.34564

x_yrsaft5 <- as.data.frame(effects_yrsaftloa5)

model5_plot <- ggplot() +
 geom_point(data=vmperqmt, aes(yrsaftloa, log(vmtot)))+
 geom_point(data=x_yrsaft5, aes(x=yrsaftloa, y=fit), color="black")+
 geom_line(data=x_yrsaft5, aes(x=yrsaftloa, y=fit), color="black")+
 geom_ribbon(data= x_yrsaft5, aes(x=yrsaftloa, ymin=lower, ymax=upper), alpha=0.2, fill="gray")+
 labs(x=" Years After LOA", y="Total VMs", title="Predictive Total VMs")+ theme_bw()

model5_plot


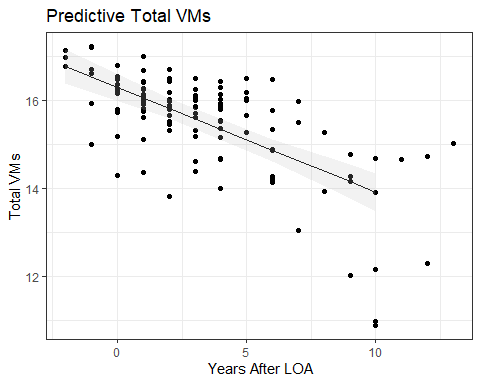


###Model VMPerMin


sjPlot::tab_model(model6,
 show.re.var= TRUE,
 pred.labels =c("(Intercept)", "Years After Loss of Ambulation"),
 dv.labels= "Effects of Time on VMs Per Minute")

Effects of Time on VMs Per Minute

Predictors

Estimates

CI

p

(Intercept)

7.12

6.89 – 7.35

<0.001

Years After Loss of Ambulation

-0.19

-0.24 – -0.15

<0.001

Random Effects

σ2

0.13

τ00 subjectid

0.38

ICC

0.74

N subjectid

65

Observations

116

Marginal R2 / Conditional R2

0.438 / 0.856

effects_yrsaftloa6 <- effects::effect(term= "yrsaftloa", mod=model6)
summary(effects_yrsaftloa6)

##
## yrsaftloa effect
## yrsaftloa
## -2 2 6 9 10
## 7.507862 6.727908 5.947954 5.362989 5.168000
##
## Lower 95 Percent Confidence Limits
## yrsaftloa
## -2 2 6 9 10
## 7.206034 6.547572 5.743492 5.057534 4.823108
##
## Upper 95 Percent Confidence Limits
## yrsaftloa
## -2 2 6 9 10
## 7.809690 6.908244 6.152417 5.668443 5.512893

x_yrsaft6 <- as.data.frame(effects_yrsaftloa6)

model6_plot <- ggplot() +
 geom_point(data=vmperqmt, aes(yrsaftloa, log(vmpermin)))+
 geom_point(data=x_yrsaft6, aes(x=yrsaftloa, y=fit), color="black")+
 geom_line(data=x_yrsaft6, aes(x=yrsaftloa, y=fit), color="black")+
 geom_ribbon(data= x_yrsaft6, aes(x=yrsaftloa, ymin=lower, ymax=upper), alpha=0.2, fill="gray")+
 labs(x=" Years After LOA", y="VMs Per Minute", title="Predictive VMs Per Minute") + theme_bw()

model6_plot


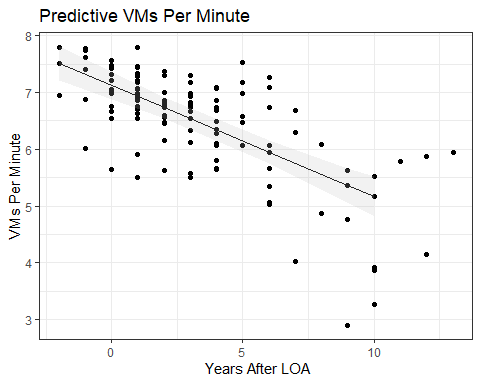


###Model VMPerDay


sjPlot::tab_model(model7,
 show.re.var= TRUE,
 pred.labels =c("(Intercept)", "Years After Loss of Ambulation"),
 dv.labels= "Effects of Time on VMs Per Day")

Effects of Time on VMs Per Day

Predictors

Estimates

CI

p

(Intercept)

14.38

14.14 – 14.62

<0.001

Years After Loss of Ambulation

-0.20

-0.25 – -0.16

<0.001

Random Effects

σ2

0.14

τ00 subjectid

0.41

ICC

0.74

N subjectid

65

Observations

116

Marginal R2 / Conditional R2

0.442 / 0.855

effects_yrsaftloa7 <- effects::effect(term= "yrsaftloa", mod=model7)
summary(effects_yrsaftloa7)

##
## yrsaftloa effect
## yrsaftloa
## -2 2 6 9 10
## 14.78355 13.96747 13.15139 12.53933 12.33531
##
## Lower 95 Percent Confidence Limits
## yrsaftloa
## -2 2 6 9 10
## 14.46980 13.78020 12.93896 12.22175 11.97669
##
## Upper 95 Percent Confidence Limits
## yrsaftloa
## -2 2 6 9 10
## 15.09729 14.15473 13.36382 12.85690 12.69393

x_yrsaft7 <- as.data.frame(effects_yrsaftloa7)

model7_plot <- ggplot() +
 geom_point(data=vmperqmt, aes(yrsaftloa, log(vmperday)))+
 geom_point(data=x_yrsaft7, aes(x=yrsaftloa, y=fit), color="black")+
 geom_line(data=x_yrsaft7, aes(x=yrsaftloa, y=fit), color="black")+
 geom_ribbon(data= x_yrsaft7, aes(x=yrsaftloa, ymin=lower, ymax=upper), alpha=0.2, fill="gray")+
 labs(x=" Years After LOA", y="VMs Per Day", title="Predictive VMs Per Day") + theme_bw()

model7_plot


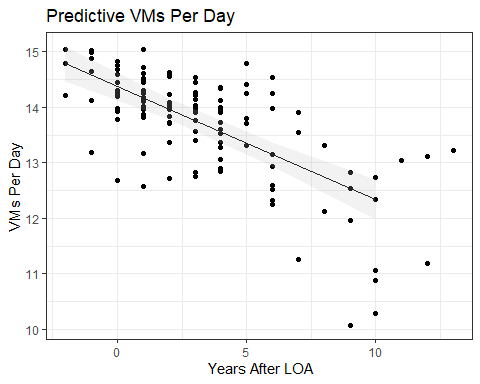


cowplot::plot_grid(p1, p2, p3, p4, labels = c('A', 'B', 'C', 'D'), label_size= 12)


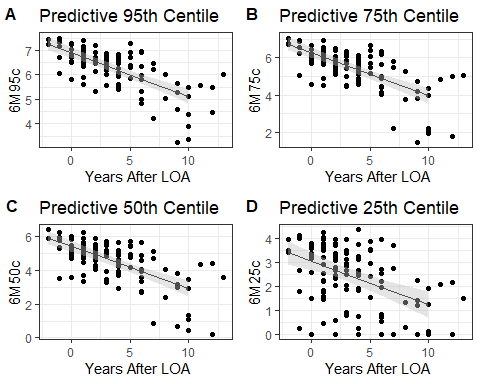

Supplement: 6 [file NIHMS2187307-supplement-6.docx]
